# Supplementary material for: Efficacy of Aedes aegypti control by indoor Ultra Low Volume (ULV) insecticide spraying in Iquitos, Peru
Source: PLoS Negl Trop Dis. 2018 Apr 6;12(4):e0006378. doi: 10.1371/journal.pntd.0006378 (PMC5906025; doi:10.1371/journal.pntd.0006378)
Supplement: S1 Appendix — (PDF) [file pntd.0006378.s001.pdf]

Supporting Information: All Figures & Tables

Efficacy of *Aedes aegypti* control by indoor Ultra Low Volume (ULV) insecticide spraying in Iquitos, Peru. CE Gunning, K Okamoto, H Astete, GM Vasquez, EB Erhardt, C Del Aguila, R Pinedo, R Cardenas, C Pacheco4, E Chalco, H Rodriguez-Ferruci, TW Scott, AL Lloyd, F Gould and AC Morrison. PLoS Negl Trop Dis. 2018. Data available at `https://doi.org/10.5061/dryad.160023v`

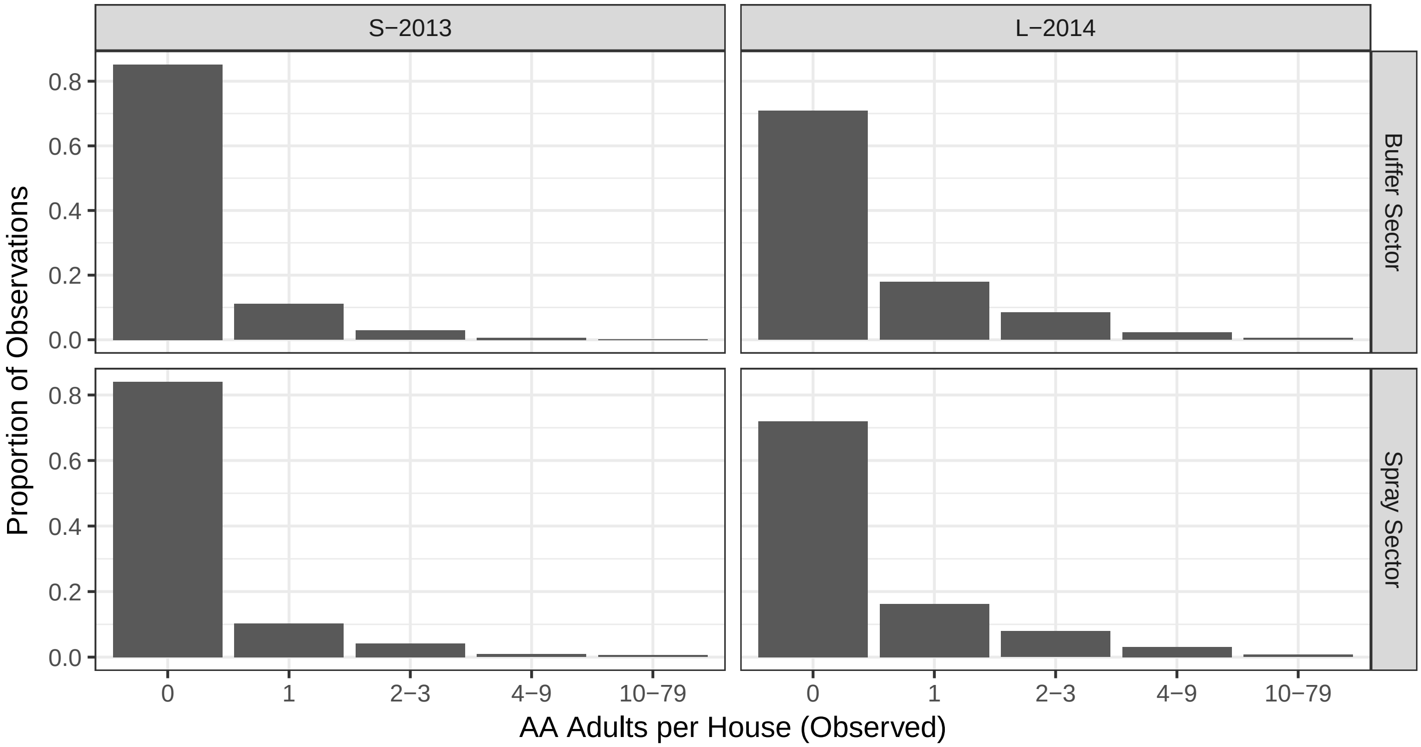

Figure S1. Histogram of AA/HSE at baseline (C1). Rows show treatment sector. X-axis is sqrt-scaled. The majority of house surveys find no adults.

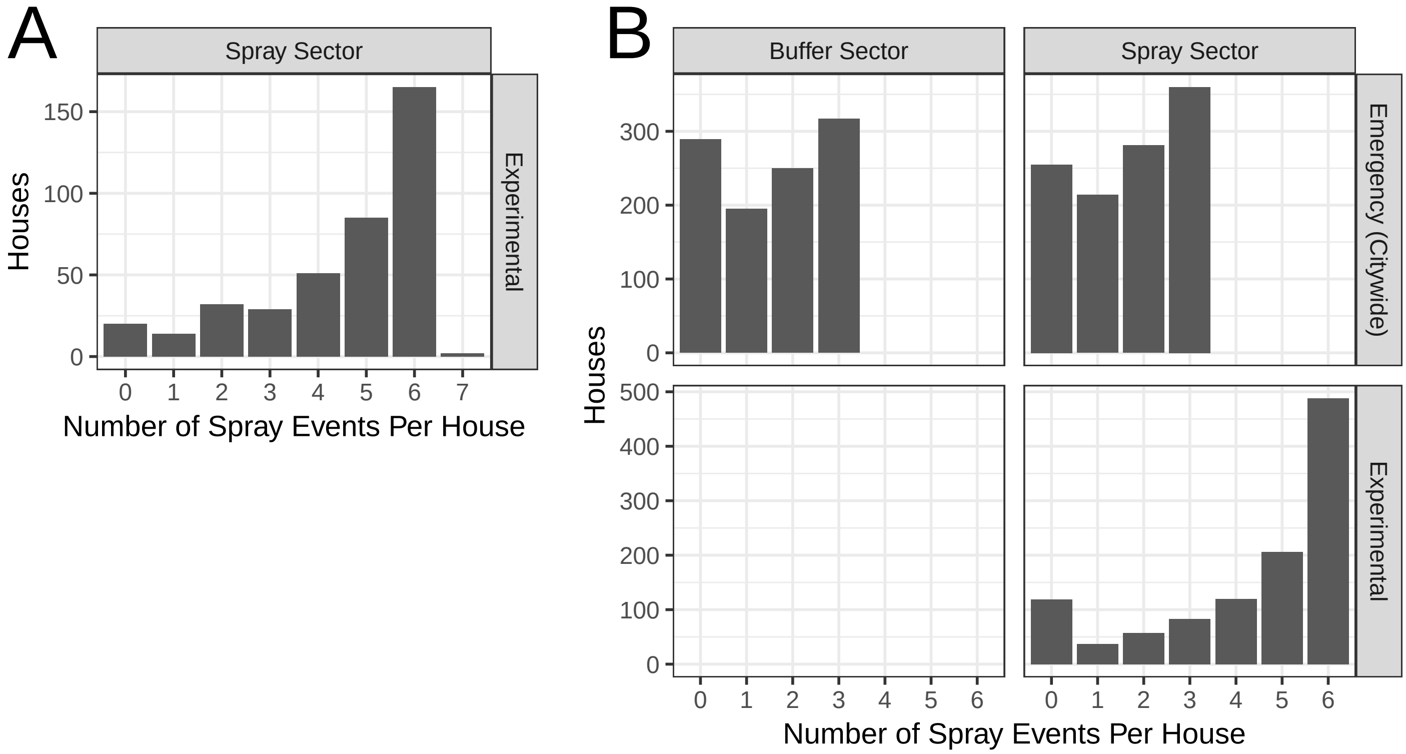

Figure S2. Summary of spray coverage in S-2013 (**A**) and L-2014 (**B**). In both years, most houses were sprayed in at least 5 out of 6 spray cycles, while a small number of houses were never sprayed. In L-2014, experimental spray coverage was much higher than emergency (citywide) spray coverage.

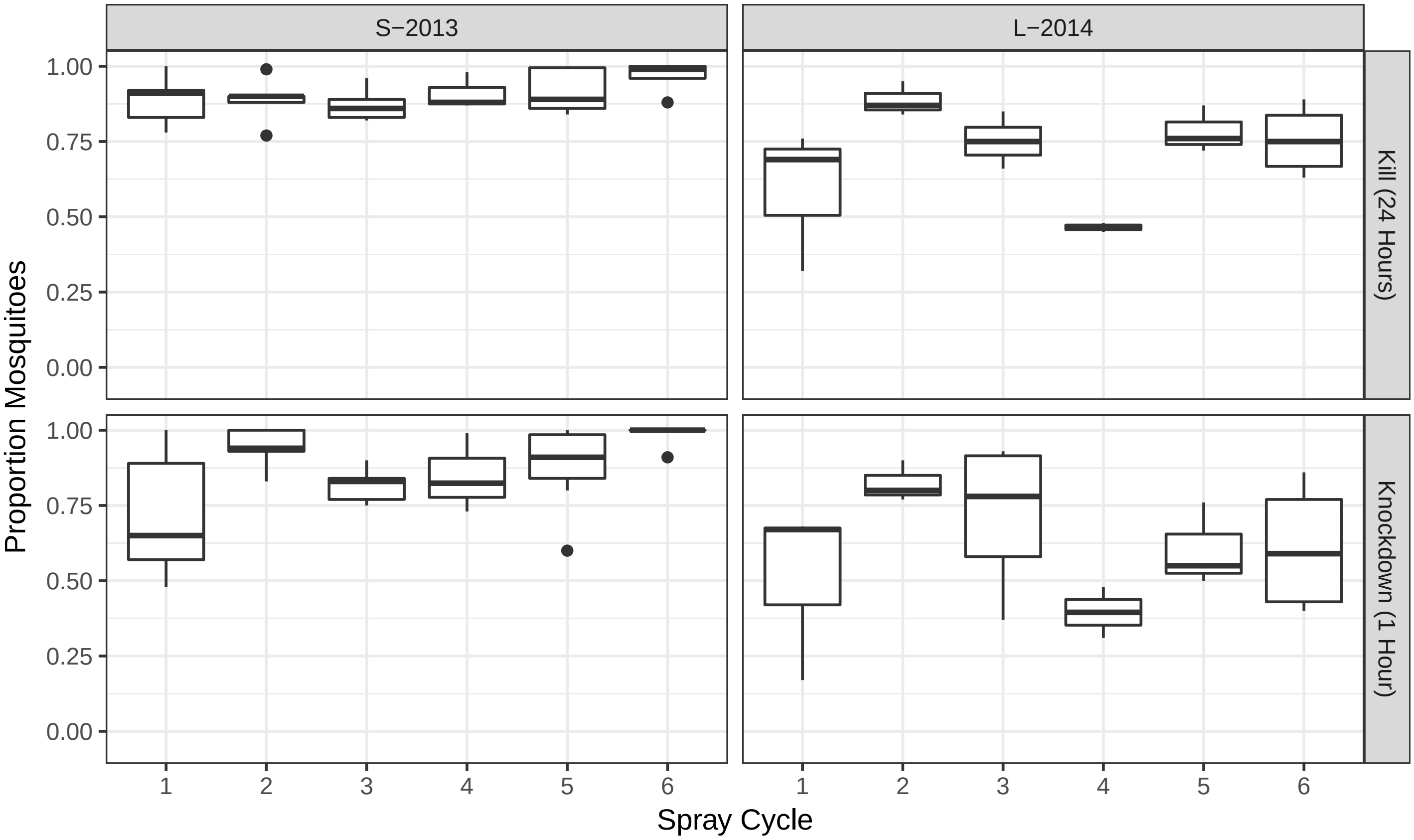

Figure S3. Boxplot of control cage house means: 25 adults per cage, 4 cages per house, approx 5 houses per spray cycle. Insects were from a laboratory colony (one colony per year).

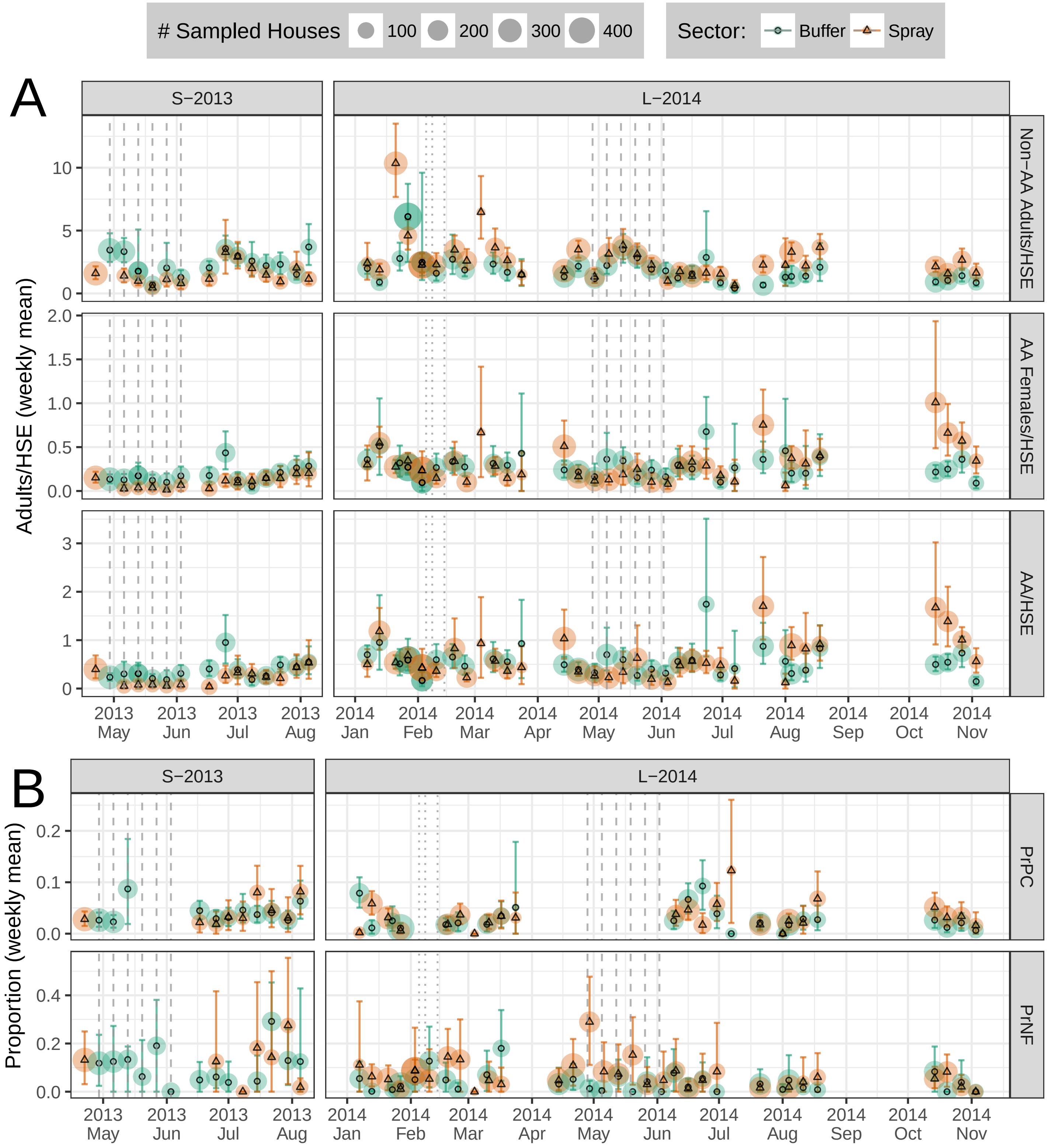

Figure S4. Time series of survey results, aggregated by week. X-axis shows week start date. Color and line-type shows treatment sector (orange triangle: Spray Sector). Point size shows number of surveyed houses. Vertical lines show approximate spray dates: dashed, experimental spraying (spray sector only); dotted, citywide spraying (Feb 2014, all sectors). Vertical colored bars show bootstrap 95% CI (1e+04 draws per circuit). **A:** Adult surveys. **B:** Container (PrPC) and parity (PrNF) surveys.

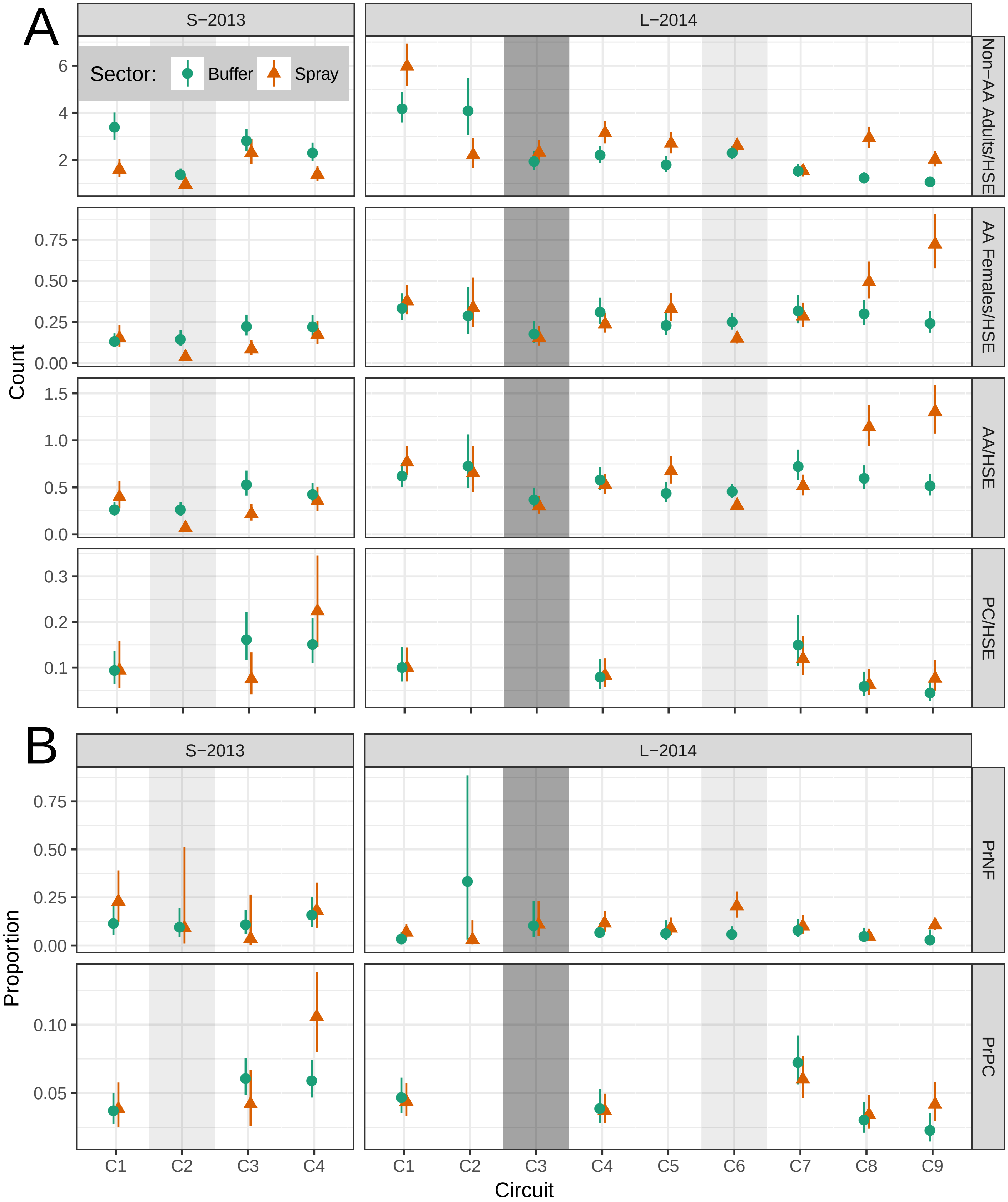

Figure S5. Model results, as in Fig 4. All models include fixed effects of sector and circuit, with a separate model for each year. **A, Counts:** negative binomial GLM (NB-GLM). **B, Proportions:** logistic GLM (L-GLM). Breteau Index (BI) = 100× PC/HSE. See also Tables S2-S10B.

Experiment	Circuit	Weeks	Treatment	Houses	Surveys	Full Surveys	Buffer	Spray
S-2013	C1	01-04		943	944	863	613	331
S-2013	C2	03-07	Exper. spray	679	983	0	603	380
S-2013	C3	09-12		935	949	885	618	331
S-2013	C4	13-16		930	967	882	614	353
L-2014	C1	01-04		1470	1473	1289	729	744
L-2014	C2	04-05		430	430	0	203	227
L-2014	C3	05-06	Citywide spray	792	848	0	411	437
L-2014	C4	07-12		1452	1500	1359	704	796
L-2014	C5	15-16		1206	1212	0	567	645
L-2014	C6	17-21	Exper. spray	1646	2502	0	1202	1300
L-2014	C7	22-27		1287	1319	1147	610	709
L-2014	C8	29-33		1461	1482	1267	720	762
L-2014	C9	41-44		1339	1358	1125	664	694

Table S1. Observation counts by Circuit. *Weeks*: Week number from experiment start. *Houses*: number of unique houses surveyed. *Surveys*: total surveys (either adult, or combined adult and immature). *Full Surveys*: surveys where both adult and immatures were surveyed. *Buffer, Spray*: surveys in buffer and spray sector, respectively.

Experiment	Circuit	Weeks	Treatment	Ratio	SE	p.value
S-2013	C1	01-04		1.52	0.31	**0.0395**
S-2013	C2	03-07	Exper. spray	0.26	0.07	**4.16e-07**
S-2013	C3	09-12		0.41	0.09	**2.19e-05**
S-2013	C4	13-16		0.84	0.16	0.357
L-2014	C1	01-04		1.24	0.16	0.09
L-2014	C2	04-05		0.90	0.21	0.659
L-2014	C3	05-06	Citywide spray	0.82	0.15	0.284
L-2014	C4	07-12		0.91	0.12	0.474
L-2014	C5	15-16		1.54	0.23	**0.0034**
L-2014	C6	17-21	Exper. spray	0.67	0.07	**0.00028**
L-2014	C7	22-27		0.71	0.10	**0.0132**
L-2014	C8	29-33		1.91	0.24	**2.72e-07**
L-2014	C9	41-44		2.53	0.34	**2.67e-12**

Table S2. Comparison between sectors (within time): Ratio of AA/HSE in spray sector relative to buffer sector (spray/buffer). **Bold p.values**: significant difference between sectors. In both years, the spray sector starts with more adults per house, and spraying reduces AA/HSE relative to buffer sectors. As in Table S3, the effects of spraying are most pronounced in 2013. See also Fig. 4A.

Experiment	Circuit	Weeks	Treatment	Ratio	SE	p.value
S-2013	C2	03-07	Exper. spray	0.17	0.05	**1.24e-09**
S-2013	C3	09-12		0.55	0.13	**0.0351**
S-2013	C4	13-16		0.89	0.20	0.944
L-2014	C2	04-05		0.85	0.16	0.979
L-2014	C3	05-06	Citywide spray	0.39	0.06	**4.87e-08**
L-2014	C4	07-12		0.69	0.09	**0.0251**
L-2014	C5	15-16		0.88	0.12	0.954
L-2014	C6	17-21	Exper. spray	0.40	0.05	**8.97e-14**
L-2014	C7	22-27		0.67	0.09	**0.0173**
L-2014	C8	29-33		1.49	0.18	**0.0103**
L-2014	C9	41-44		1.70	0.21	**0.000172**

Table S3. Comparison between times (within spray sector): Ratio of AA/HSE relative to baseline (C1, spray sector only). **Bold p.values**: significant difference from baseline circuit. In both years, spraying reduces AA/HSE relative to baseline (C1). The effects of spraying are most pronounced in S-2013, but are short-lived in both years. See also Fig. 4A.

Experiment	Contrast	Ratio	SE	p.value
S-2013	No Prior Spray / Prior Spray	1.58	1.11	0.764
L-2014	No Prior Spray / Prior Spray	2.01	0.62	**0.0472**
L-2014	Timing Unclear / Prior Spray	0.71	0.26	0.566

Table S4. Comparison between spray status (whether house was sprayed prior to survey): Ratio of AA/HSE in houses that were either sprayed or not spray prior to surveying (no prior spray / prior spray). **Bold p.values**: In L-2014, houses without prior spraying yielded significantly more adults than houses with prior spraying. In S-2013, most houses were sprayed in the prior week. In L-2014, the exact date of spraying was uncertain for a small number of houses. See Table S5 for details.

Experiment	Spray.status	nObs	Group	Est	SE	95% CI
S-2013	Prior Spray	311	b	0.06	0.02	0.03-0.14
S-2013	No Prior Spray	59	ab	0.10	0.06	0.02-0.54
L-2014	Prior Spray	889	a	0.28	0.04	0.19-0.40
L-2014	No Prior Spray	205	a	0.56	0.15	0.27-1.15
L-2014	Timing Unclear	164	ab	0.20	0.07	0.08-0.48

Table S5. Effect of prior spray on AA/HSE. A single model (NB-GLM) includes both experiment year and spray status as predictors. *Group:* significance groups (Tukey HSD) compare among all rows. Only house surveys in the spray sector during experimental spraying are included (i.e., S-2013 C2 and L-2014 C6). Not all sprayed houses were subsequently surveyed. The average interval between each house's spray application and survey was shorter in L-2014 (median 2 days) than S-2013 (median 7 days). See also Table S4.

Circuit	Weeks	Treatment	Sector	nObs	Group	Est	SE	95% CI
C1	01-04		Buffer	613	ab	0.26	0.03	0.19-0.37
C2	03-07		Buffer	603	ab	0.26	0.03	0.18-0.37
C3	09-12		Buffer	618	c	0.53	0.06	0.39-0.72
C4	13-16		Buffer	614	a c	0.42	0.05	0.31-0.58
C1	01-04		Spray	331	abc	0.40	0.06	0.26-0.61
C2	03-07	Exper. spray	Spray	380	d	0.07	0.02	0.04-0.13
C3	09-12		Spray	331	b	0.22	0.04	0.13-0.35
C4	13-16		Spray	353	abc	0.35	0.06	0.23-0.54

Table S6A. *Ae. aegypti* adults per house (AA/HSE), 2013. Model estimates by circuit and treatment sector. Horizontal line separates treatment sectors; significance groups (Tukey HSD) compare among all rows. See Fig. 4A for model description.

Circuit	Weeks	Treatment	Sector	nObs	Group	Est	SE	95% CI
C1	01-04		Buffer	729	abcd	0.62	0.06	0.47-0.81
C2	04-05		Buffer	203	abcdef	0.72	0.12	0.43-1.21
C3	05-06	Citywide spray	Buffer	411	a gh	0.37	0.05	0.25-0.55
C4	07-12		Buffer	704	abcd	0.58	0.05	0.44-0.77
C5	15-16		Buffer	567	abc gh	0.44	0.05	0.31-0.61
C6	17-21		Buffer	1202	a c g	0.46	0.03	0.36-0.57
C7	22-27		Buffer	610	b d	0.72	0.07	0.54-0.97
C8	29-33		Buffer	720	abcd	0.60	0.06	0.45-0.79
C9	41-44		Buffer	664	abcd g	0.52	0.05	0.38-0.69
C1	01-04		Spray	744	de	0.77	0.07	0.59-1.00
C2	04-05		Spray	227	abcde	0.65	0.11	0.40-1.07
C3	05-06	Citywide spray	Spray	437	gh	0.30	0.04	0.20-0.45
C4	07-12		Spray	796	abcd g	0.53	0.05	0.40-0.69
C5	15-16		Spray	645	bcd	0.67	0.07	0.50-0.90
C6	17-21	Exper. spray	Spray	1300	h	0.31	0.02	0.24-0.39
C7	22-27		Spray	709	abcd g	0.51	0.05	0.39-0.68
C8	29-33		Spray	762	ef	1.14	0.10	0.89-1.47
C9	41-44		Spray	694	f	1.31	0.12	1.01-1.70

Table S6B. *Ae. aegypti* adults per house (AA/HSE), 2014. See Table S6A for details.

Circuit	Weeks	Treatment	Sector	nObs	Group	Est	SE	95% CI
C1	01-04		Buffer	613	ab	0.15	0.01	0.11-0.19
C2	03-07		Buffer	603	ab	0.15	0.01	0.11-0.19
C3	09-12		Buffer	618	a c	0.21	0.02	0.17-0.26
C4	13-16		Buffer	614	c	0.23	0.02	0.19-0.28
C1	01-04		Spray	331	abc	0.16	0.02	0.11-0.22
C2	03-07	Exper. spray	Spray	380	d	0.06	0.01	0.03-0.10
C3	09-12		Spray	331	b	0.13	0.02	0.08-0.19
C4	13-16		Spray	353	abc	0.17	0.02	0.12-0.23

Table S7A. Proportion *Ae. aegypti* adult-infested houses (PrIH), 2013. Model estimates by circuit and treatment sector. Horizontal line separates treatment sectors; significance groups (Tukey HSD) compare among all rows. See Fig. 4B for model description.

Circuit	Weeks	Treatment	Sector	nObs	Group	Est	SE	95% CI
C1	01-04		Buffer	729	ab	0.31	0.02	0.26-0.36
C2	04-05		Buffer	203	abcd	0.33	0.03	0.24-0.43
C3	05-06	Citywide spray	Buffer	411	ef	0.16	0.02	0.11-0.22
C4	07-12		Buffer	704	abc g	0.26	0.02	0.22-0.32
C5	15-16		Buffer	567	c e g	0.22	0.02	0.17-0.28
C6	17-21		Buffer	1202	c e g	0.21	0.01	0.18-0.25
C7	22-27		Buffer	610	abc g	0.27	0.02	0.22-0.33
C8	29-33		Buffer	720	c e g	0.22	0.02	0.18-0.27
C9	41-44		Buffer	664	abc g	0.26	0.02	0.21-0.32
C1	01-04		Spray	744	a d	0.34	0.02	0.29-0.39
C2	04-05		Spray	227	abcd g	0.29	0.03	0.21-0.39
C3	05-06	Citywide spray	Spray	437	e g	0.18	0.02	0.13-0.24
C4	07-12		Spray	796	bc e g	0.23	0.01	0.19-0.28
C5	15-16		Spray	645	abc	0.28	0.02	0.23-0.33
C6	17-21	Exper. spray	Spray	1300	f	0.11	0.01	0.09-0.14
C7	22-27		Spray	709	c e g	0.22	0.02	0.18-0.27
C8	29-33		Spray	762	a d	0.34	0.02	0.29-0.40
C9	41-44		Spray	694	d	0.41	0.02	0.36-0.47

Table S7B. Proportion *Ae. aegypti* adult-infested houses (PrIH), 2014. See Table S7A for details.

Circuit	Weeks	Treatment	Sector	nObs	Group	Est	SE	95% CI
C1	01-04		Buffer	58	a	0.11	0.04	0.05-0.25
C2	03-07		Buffer	52	a	0.10	0.03	0.04-0.22
C3	09-12		Buffer	73	a	0.11	0.03	0.05-0.21
C4	13-16		Buffer	92	a	0.16	0.03	0.09-0.28
C1	01-04		Spray	32	a	0.23	0.06	0.10-0.43
C2	03-07	Exper. spray	Spray	9	a	0.09	0.09	0.01-0.64
C3	09-12		Spray	23	a	0.04	0.04	0.00-0.37
C4	13-16		Spray	34	a	0.18	0.05	0.08-0.37

Table S8A. Proportion nulliparouous *Ae. aegypti* females (PrNF), 2013. Model estimates by circuit and treatment sector. Horizontal line separates treatment sectors; significance groups (Tukey HSD) compare among all rows. See also Fig. S5.

Circuit	Weeks	Treatment	Sector	nObs	Group	Est	SE	95% CI
C1	01-04		Buffer	144	a	0.03	0.01	0.01-0.09
C2	04-05		Buffer	3	ab	0.33	0.27	0.01-0.95
C3	05-06	Citywide spray	Buffer	39	ab	0.10	0.04	0.03-0.29
C4	07-12		Buffer	120	a	0.07	0.02	0.03-0.14
C5	15-16		Buffer	76	ab	0.06	0.02	0.02-0.17
C6	17-21		Buffer	155	a	0.06	0.01	0.03-0.12
C7	22-27		Buffer	93	ab	0.08	0.02	0.04-0.16
C8	29-33		Buffer	95	a	0.05	0.01	0.02-0.11
C9	41-44		Buffer	93	a	0.03	0.01	0.01-0.12
C1	01-04		Spray	155	a	0.07	0.02	0.03-0.13
C2	04-05		Spray	39	ab	0.03	0.02	0.00-0.21
C3	05-06	Citywide spray	Spray	47	ab	0.11	0.04	0.04-0.29
C4	07-12		Spray	106	ab	0.12	0.02	0.06-0.21
C5	15-16		Spray	101	ab	0.09	0.02	0.04-0.17
C6	17-21	Exper. spray	Spray	95	b	0.20	0.03	0.13-0.31
C7	22-27		Spray	96	ab	0.10	0.02	0.05-0.19
C8	29-33		Spray	161	a	0.05	0.01	0.02-0.09
C9	41-44		Spray	197	ab	0.11	0.01	0.07-0.16

Table S8B. Proportion nulliparouous *Ae. aegypti* females (PrNF), 2014. See Table S8A for details.

Circuit	Weeks	Sector	nObs	Group	Est	SE	95% CI
C1	01-04	Buffer	565	a	0.09	0.02	0.060-0.147
C3	09-12	Buffer	590	ab	0.16	0.02	0.111-0.234
C4	13-16	Buffer	583	ab	0.15	0.02	0.103-0.221
C1	01-04	Spray	297	ab	0.09	0.02	0.051-0.175
C3	09-12	Spray	282	a	0.07	0.02	0.038-0.148
C4	13-16	Spray	268	b	0.22	0.04	0.134-0.373

Table S9A. *Ae. aegypti* Positive Containers per House (PC/HSE), 2013. Breteau index (BI) = 100× PC/HSE. Model estimates by circuit and treatment sector. Horizontal line separates treatment sectors; significance groups (Tukey HSD) compare among all rows. No container surveys were conducted during spraying. See also Fig. S5.

Circuit	Weeks	Sector	nObs	Group	Est	SE	95% CI
C1	01-04	Buffer	638	abc	0.10	0.02	0.063-0.159
C4	07-12	Buffer	606	abc	0.08	0.01	0.048-0.131
C7	22-27	Buffer	514	a	0.15	0.02	0.095-0.237
C8	29-33	Buffer	629	bc	0.06	0.01	0.034-0.102
C9	41-44	Buffer	564	b	0.04	0.01	0.023-0.084
C1	01-04	Spray	649	abc	0.10	0.02	0.064-0.158
C4	07-12	Spray	710	abc	0.08	0.01	0.052-0.132
C7	22-27	Spray	613	a c	0.12	0.02	0.076-0.186
C8	29-33	Spray	621	bc	0.06	0.01	0.037-0.108
C9	41-44	Spray	551	abc	0.08	0.01	0.045-0.130

Table S9B. *Ae. aegypti* Positive Containers per House (PC/HSE), 2014. Breteau index (BI) = 100× PC/HSE. See Table S9A for details.

Circuit	Weeks	Sector	nObs	Group	Est	SE	95% CI
C1	01-04	Buffer	565	a	0.04	0.00	0.026-0.053
C3	09-12	Buffer	590	b	0.06	0.01	0.047-0.079
C4	13-16	Buffer	583	ab	0.06	0.01	0.045-0.077
C1	01-04	Spray	297	ab	0.04	0.01	0.023-0.062
C3	09-12	Spray	282	ab	0.04	0.01	0.024-0.073
C4	13-16	Spray	268	c	0.11	0.01	0.076-0.145

Table S10A. Proportion *Ae. aegypti* Positive Containers (PrPC), 2013. Model estimates by circuit and treatment sector. Horizontal line separates treatment sectors; significance groups (Tukey HSD) compare among all rows. No container surveys were conducted during spraying. See also Fig. S5.

Circuit	Weeks	Sector	nObs	Group	Est	SE	95% CI
C1	01-04	Buffer	638	abc	0.05	0.01	0.033-0.066
C4	07-12	Buffer	606	ab	0.04	0.01	0.026-0.057
C7	22-27	Buffer	514	c	0.07	0.01	0.053-0.098
C8	29-33	Buffer	629	a	0.03	0.00	0.019-0.048
C9	41-44	Buffer	564	a	0.02	0.00	0.013-0.040
C1	01-04	Spray	649	abc	0.04	0.01	0.031-0.061
C4	07-12	Spray	710	ab	0.04	0.00	0.026-0.053
C7	22-27	Spray	613	bc	0.06	0.01	0.044-0.082
C8	29-33	Spray	621	ab	0.03	0.01	0.022-0.053
C9	41-44	Spray	551	abc	0.04	0.01	0.027-0.063

Table S10B. Proportion *Ae. aegypti* Positive Containers (PrPC), 2014. See Table S10A for details.

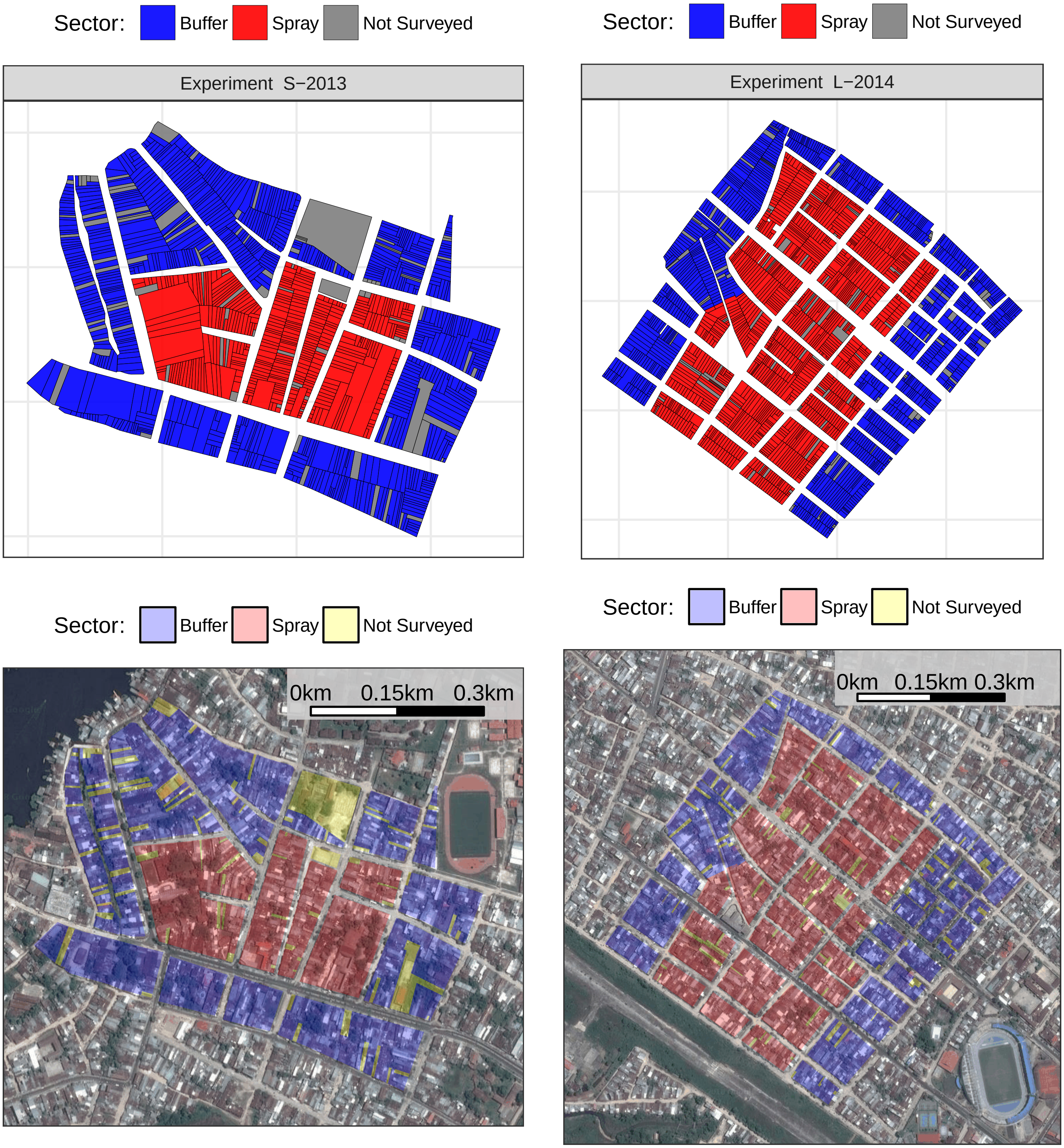

Figure S6. Maps of experimental areas, showing satellite imagery. Note the scale differs between experiments. See also Fig. 1.

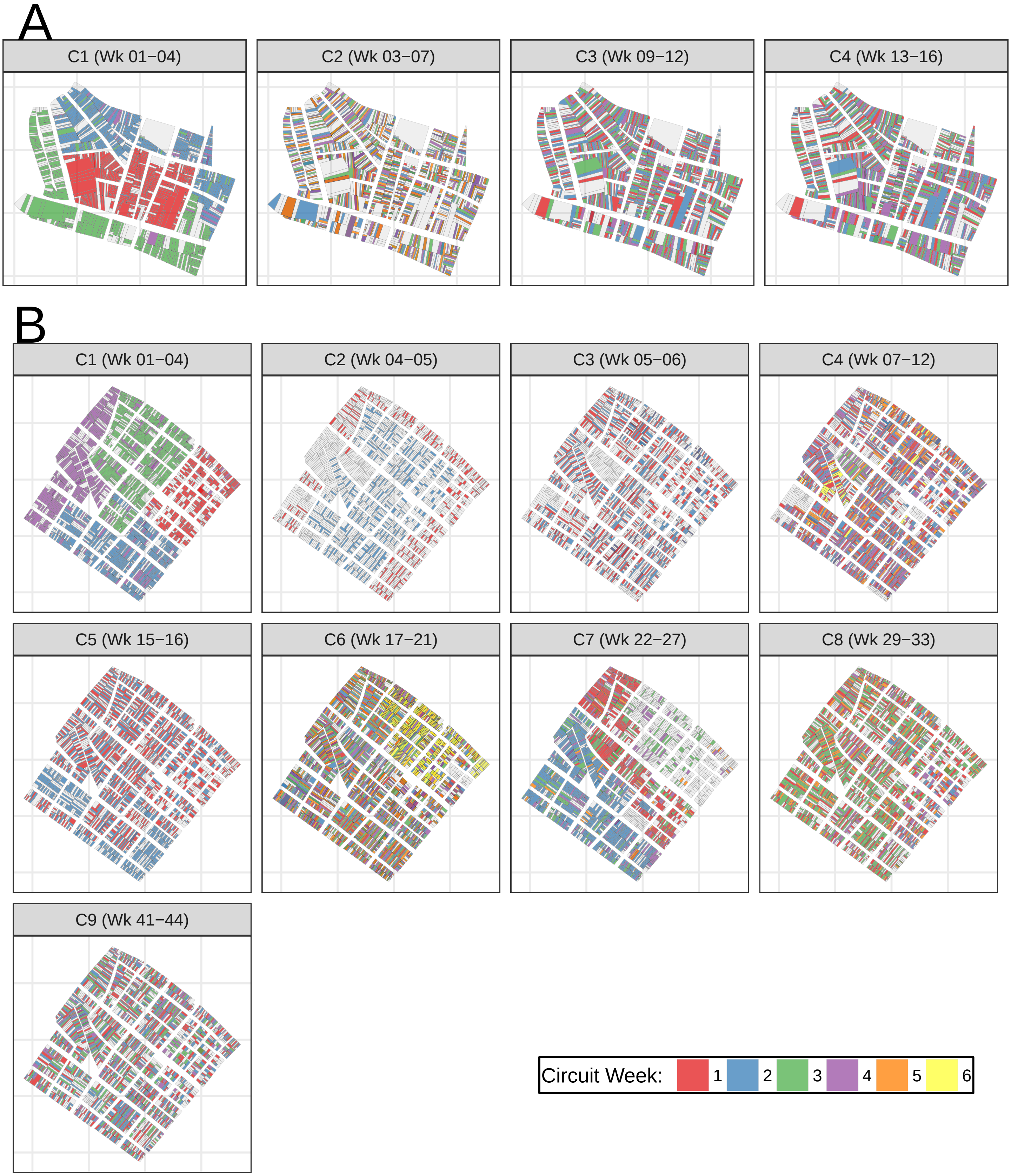

Figure S7. Maps showing survey locations by circuit (panel) and week within circuit (color). **A**: S-2013. **B**: L-2014.

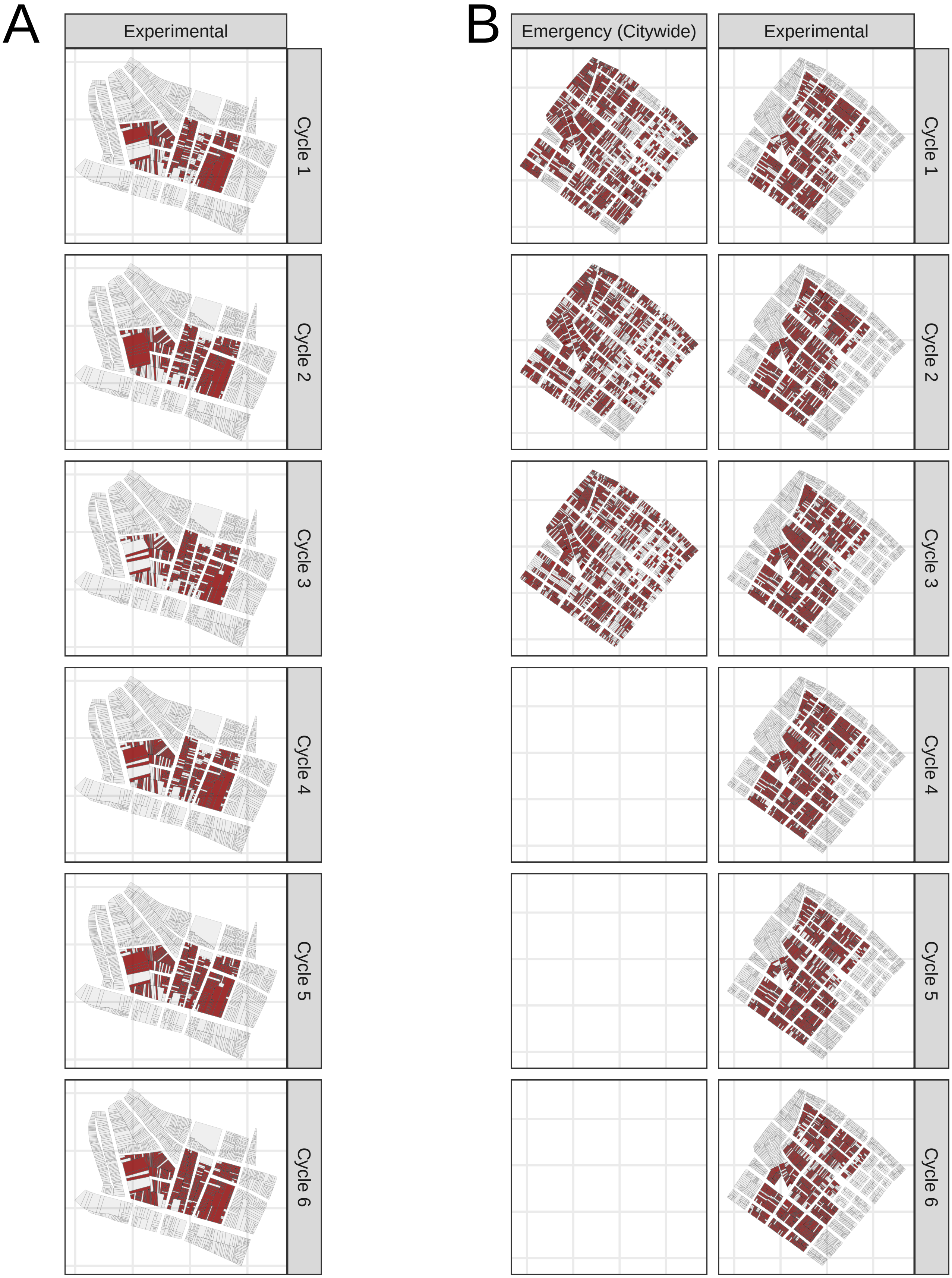

Figure S8. Maps of spray events (red) by spray cycle (rows). **A**: S-2013. **B**: L-2014. During L-2014, in addition to experimental spraying, 3 cycles of emergency citywide spraying were conducted. Note the map scale differs between **A** and **B**. See also Fig. 1.
