# Supplementary material for: Efficacy of Aedes aegypti control by indoor Ultra Low Volume (ULV) insecticide spraying in Iquitos, Peru
Source: PLoS Negl Trop Dis. 2018 Apr 6;12(4):e0006378. doi: 10.1371/journal.pntd.0006378 (PMC5906025; doi:10.1371/journal.pntd.0006378)
Supplement: S1 Fig — Rows show treatment sector. X-axis is sqrt-scaled. The majority of house surveys find no adults. (PDF) [file pntd.0006378.s002.pdf]

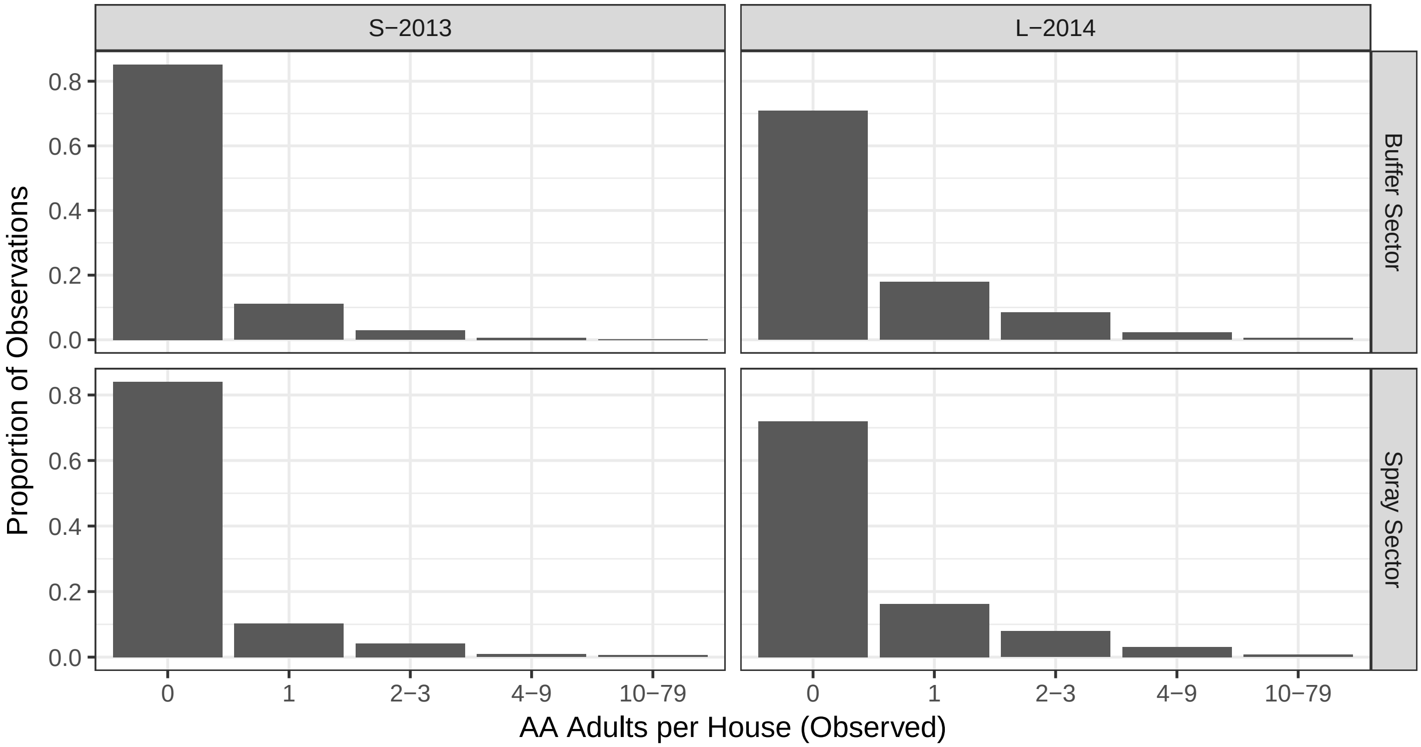

Figure S1. Histogram of AA/HSE at baseline (C1). Rows show treatment sector. X-axis is sqrt-scaled. The majority of house surveys find no adults.
