# Supplementary material for: Efficacy of Aedes aegypti control by indoor Ultra Low Volume (ULV) insecticide spraying in Iquitos, Peru
Source: PLoS Negl Trop Dis. 2018 Apr 6;12(4):e0006378. doi: 10.1371/journal.pntd.0006378 (PMC5906025; doi:10.1371/journal.pntd.0006378)
Supplement: S2 Fig — Summary of spray coverage in S-2013 (A) and L-2014 (B). In both years, most houses were sprayed in at least 5 out of 6 spray cycles, while a small number of houses were never sprayed. In L-2014, experimental spray coverage was much higher than emergency (citywide) spray coverage. (PDF) [file pntd.0006378.s003.pdf]

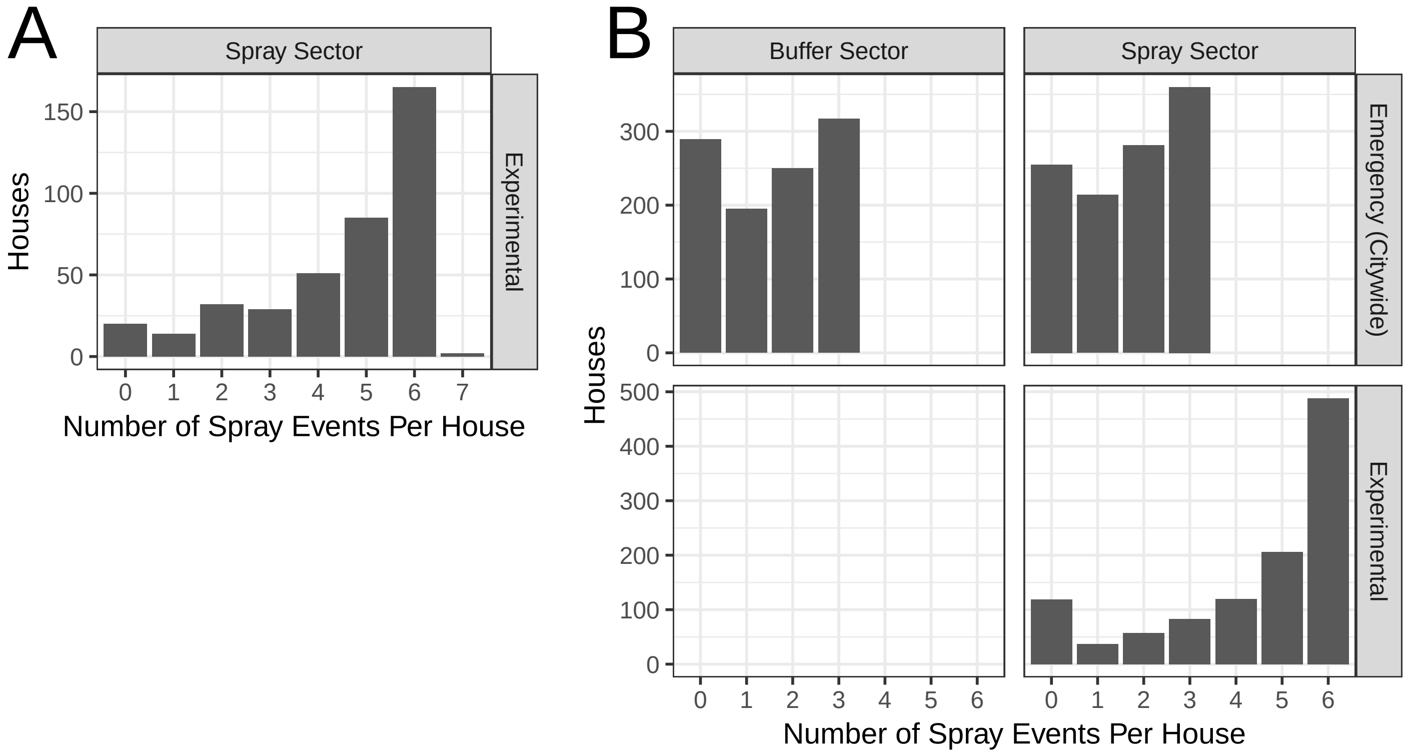

Figure S2. Summary of spray coverage in S-2013 (**A**) and L-2014 (**B**). In both years, most houses were sprayed in at least 5 out of 6 spray cycles, while a small number of houses were never sprayed. In L-2014, experimental spray coverage was much higher than emergency (citywide) spray coverage.
