# Supplementary material for: Efficacy of Aedes aegypti control by indoor Ultra Low Volume (ULV) insecticide spraying in Iquitos, Peru
Source: PLoS Negl Trop Dis. 2018 Apr 6;12(4):e0006378. doi: 10.1371/journal.pntd.0006378 (PMC5906025; doi:10.1371/journal.pntd.0006378)
Supplement: S3 Fig — Insects were from a laboratory colony (one colony per year). (PDF) [file pntd.0006378.s004.pdf]

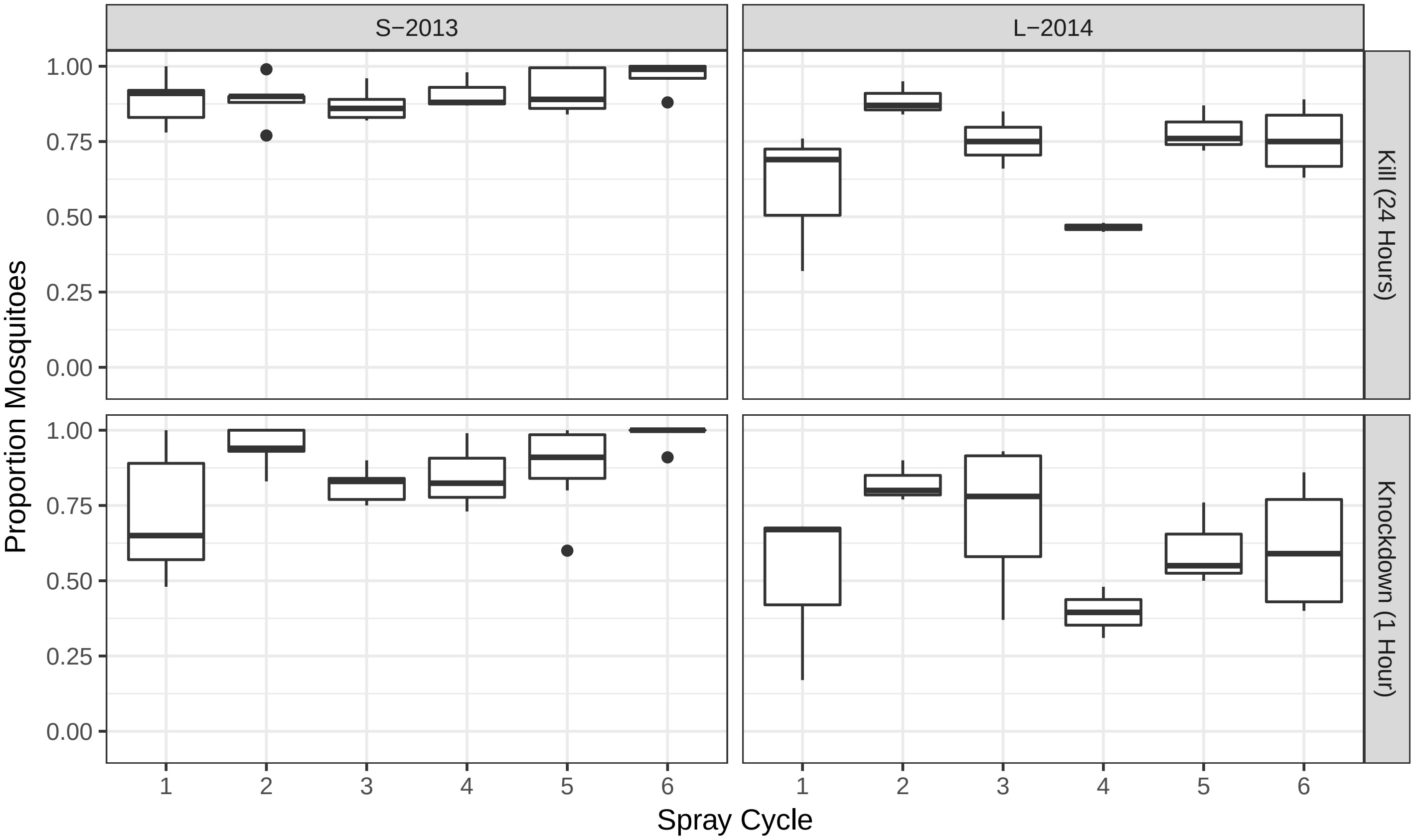

Figure S3. Boxplot of control cage house means: 25 adults per cage, 4 cages per house, approx 5 houses per spray cycle. Insects were from a laboratory colony (one colony per year).
