# Supplementary material for: Efficacy of Aedes aegypti control by indoor Ultra Low Volume (ULV) insecticide spraying in Iquitos, Peru
Source: PLoS Negl Trop Dis. 2018 Apr 6;12(4):e0006378. doi: 10.1371/journal.pntd.0006378 (PMC5906025; doi:10.1371/journal.pntd.0006378)
Supplement: S4 Fig — X-axis shows week start date. Color and line-type shows treatment sector (orange triangle: spray sector). Point size shows number of surveyed houses. Vertical lines show approximately spray dates: dashed, experimental spraying (spray sector only); dotted, citywide spraying (February 2014, all sectors). Vertical colored bars show bootstrap 95% CI (1e+04 draws per circuit). (A) Adult surveys. (B) Container (PrPC) and parity (PrNF) surveys. (PDF) [file pntd.0006378.s005.pdf]

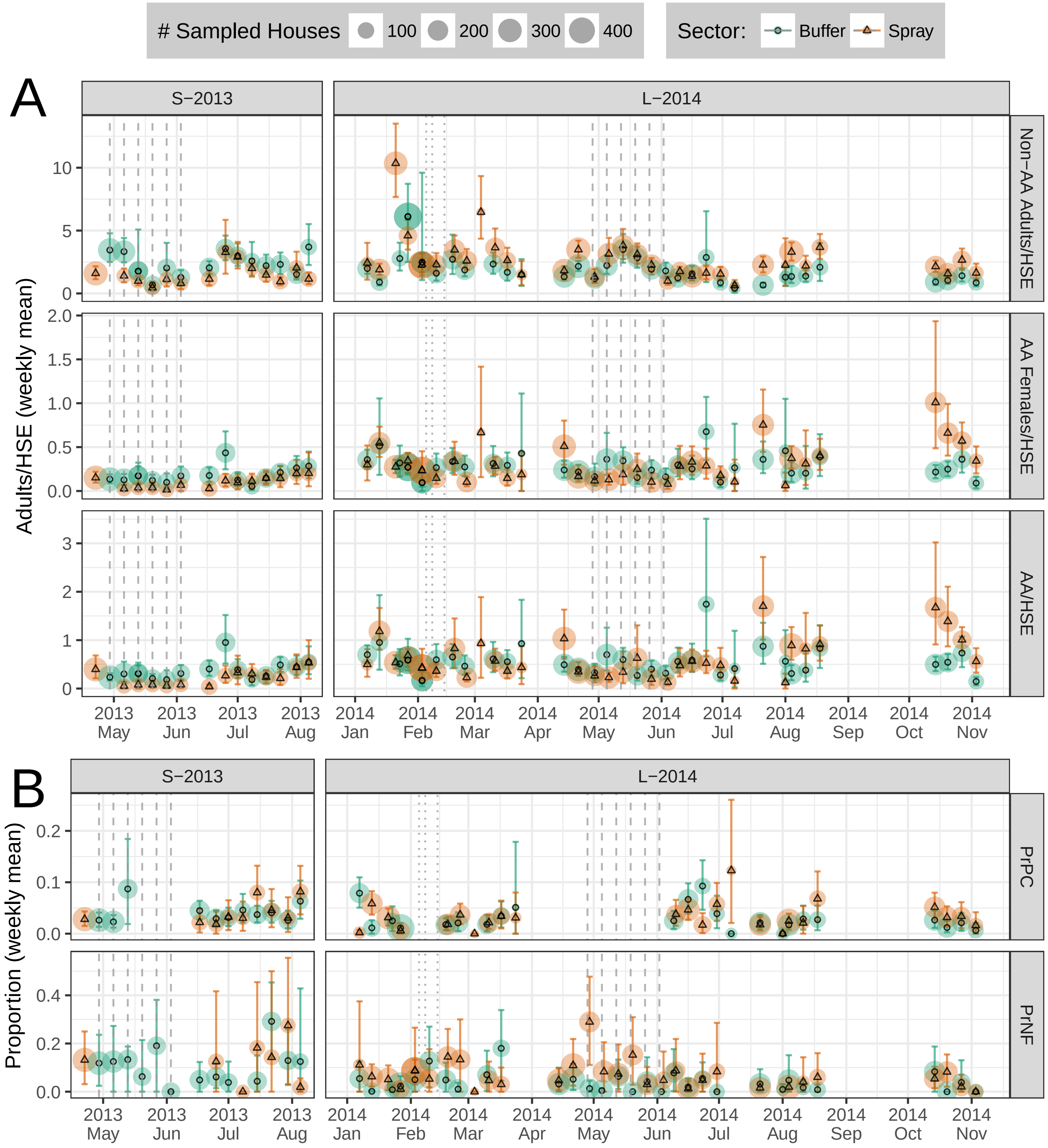

Figure S4. Time series of survey results, aggregated by week. X-axis shows week start date. Color and line-type shows treatment sector (orange triangle: Spray Sector). Point size shows number of surveyed houses. Vertical lines show approximate spray dates: dashed, experimental spraying (spray sector only); dotted, citywide spraying (Feb 2014, all sectors). Vertical colored bars show bootstrap 95% CI (1e+04 draws per circuit). **A:** Adult surveys. **B:** Container (PrPC) and parity (PrNF) surveys.
