# Supplementary material for: Efficacy of Aedes aegypti control by indoor Ultra Low Volume (ULV) insecticide spraying in Iquitos, Peru
Source: PLoS Negl Trop Dis. 2018 Apr 6;12(4):e0006378. doi: 10.1371/journal.pntd.0006378 (PMC5906025; doi:10.1371/journal.pntd.0006378)
Supplement: S5 Fig — All models include fixed effects of sector and circuit, with a separate model for each year. (A) Counts: negative binomial GLM (NB-GLM). (B) Proportions: logistic GLM (L-GLM). Note that Breteau Index (BI) = 100*PC/HSE. See also S2–S10 Tables. (PDF) [file pntd.0006378.s006.pdf]

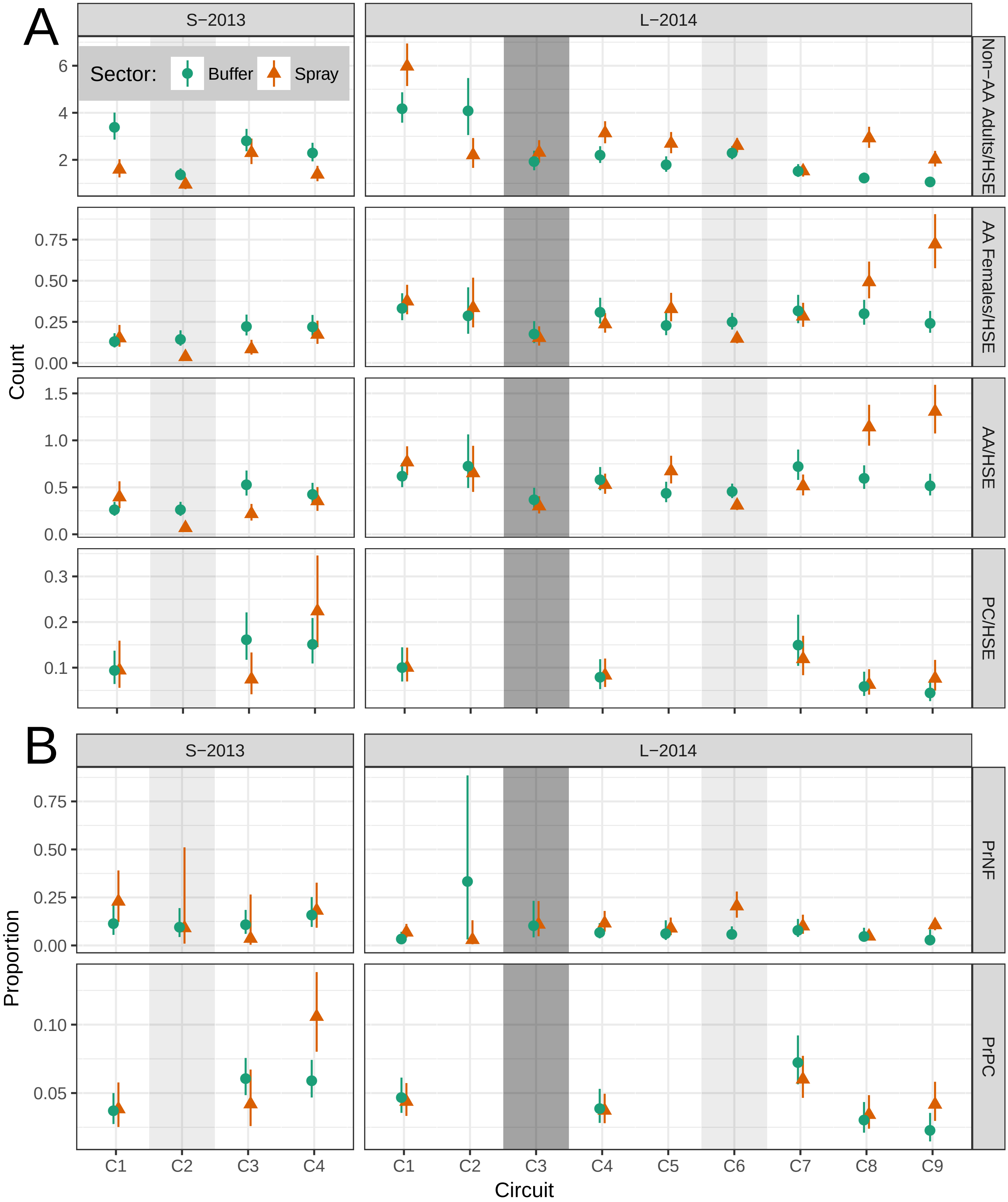

Figure S5. Model results, as in Fig 4. All models include fixed effects of sector and circuit, with a separate model for each year. **A, Counts:** negative binomial GLM (NB-GLM). **B, Proportions:** logistic GLM (L-GLM). Breteau Index (BI) = 100× PC/HSE. See also Tables S2-S10B.
