# Supplementary material for: Efficacy of Aedes aegypti control by indoor Ultra Low Volume (ULV) insecticide spraying in Iquitos, Peru
Source: PLoS Negl Trop Dis. 2018 Apr 6;12(4):e0006378. doi: 10.1371/journal.pntd.0006378 (PMC5906025; doi:10.1371/journal.pntd.0006378)
Supplement: S6 Fig — Note the scale differs between experiments. See also Fig 1. (PDF) [file pntd.0006378.s007.pdf]

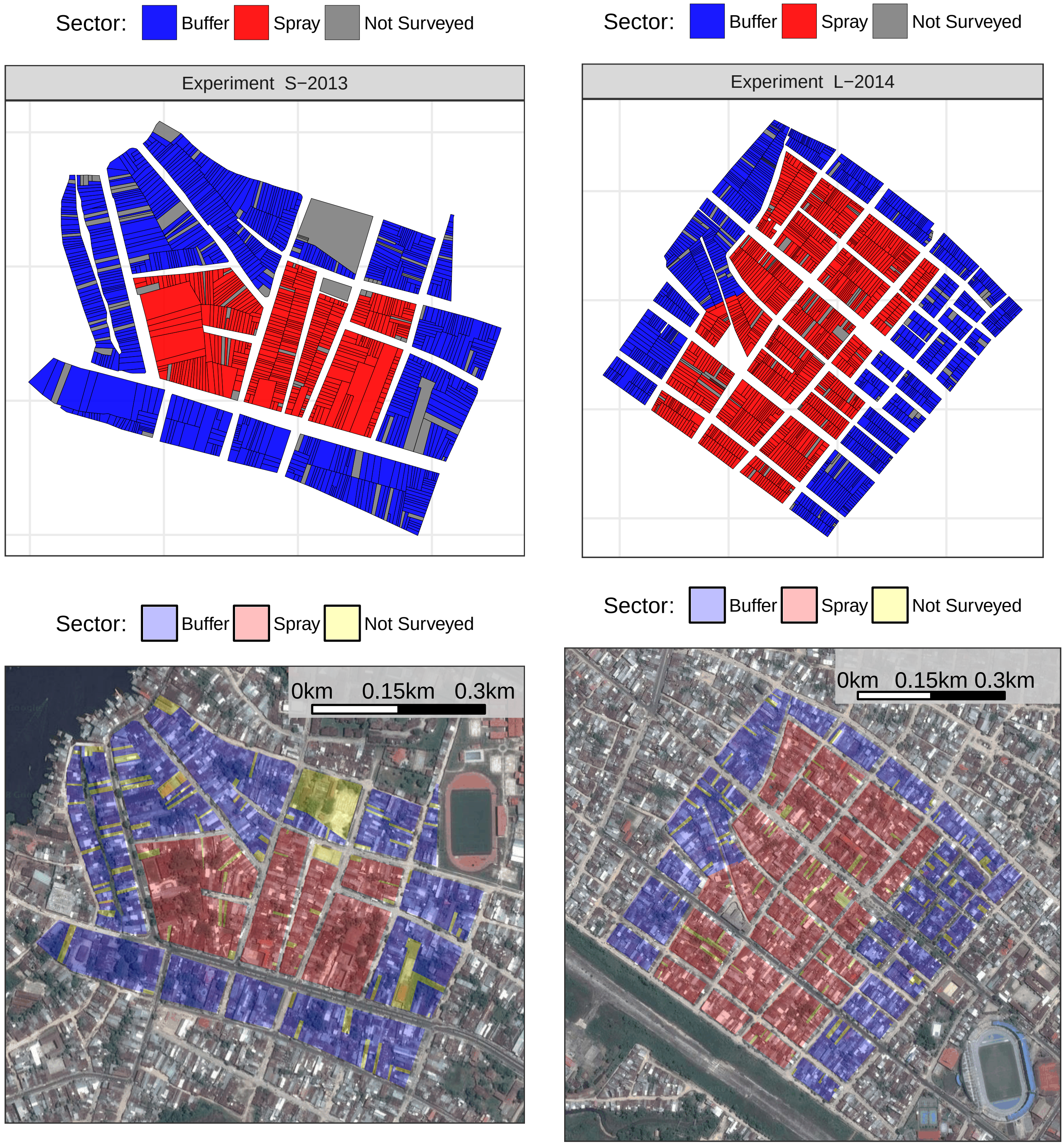

Figure S6. Maps of experimental areas, showing satellite imagery. Note the scale differs between experiments. See also Fig. 1.
