# Supplementary figures and images for: Efficacy of Aedes aegypti control by indoor Ultra Low Volume (ULV) insecticide spraying in Iquitos, Peru
Source: PLoS Negl Trop Dis. 2018 Apr 6;12(4):e0006378. doi: 10.1371/journal.pntd.0006378 (PMC5906025; doi:10.1371/journal.pntd.0006378)

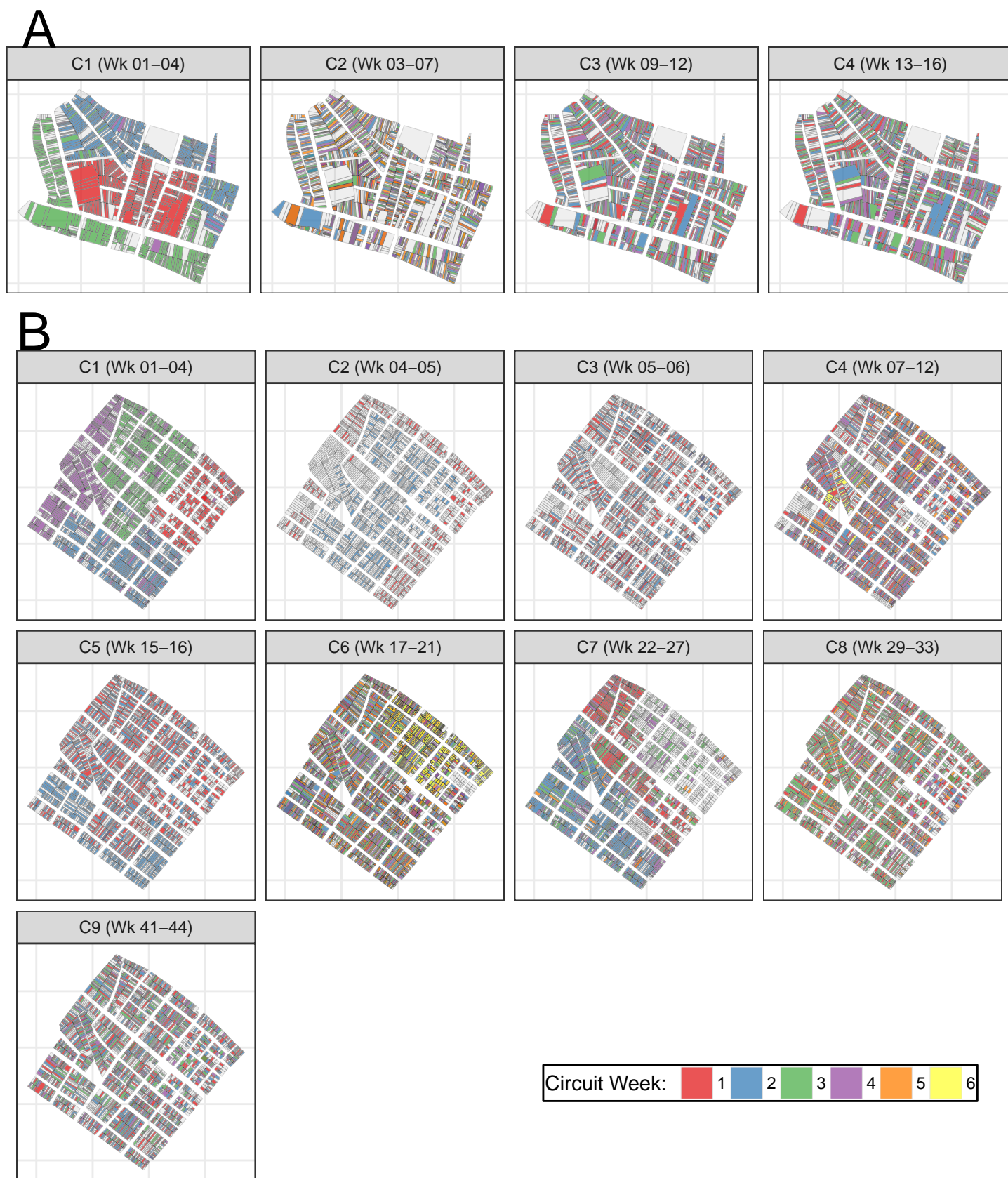

**Figure S7.** Maps showing survey locations by circuit (panel) and week within circuit (color). **A:** S-2013. **B:** L-2014.

Supplement: S7 Fig — (A) S-2013. (B) L-2014. (PDF) [file pntd.0006378.s008.pdf]
