# Supplementary material for: Efficacy of Aedes aegypti control by indoor Ultra Low Volume (ULV) insecticide spraying in Iquitos, Peru
Source: PLoS Negl Trop Dis. 2018 Apr 6;12(4):e0006378. doi: 10.1371/journal.pntd.0006378 (PMC5906025; doi:10.1371/journal.pntd.0006378)
Supplement: S8 Fig — (A) S-2013. (B) L-2014. During L-2014, in addition to experimental spraying, 3 cycles of emergency citywide spraying were conducted. Note the map scale differs between A and B. See also Fig 1. (PDF) [file pntd.0006378.s009.pdf]

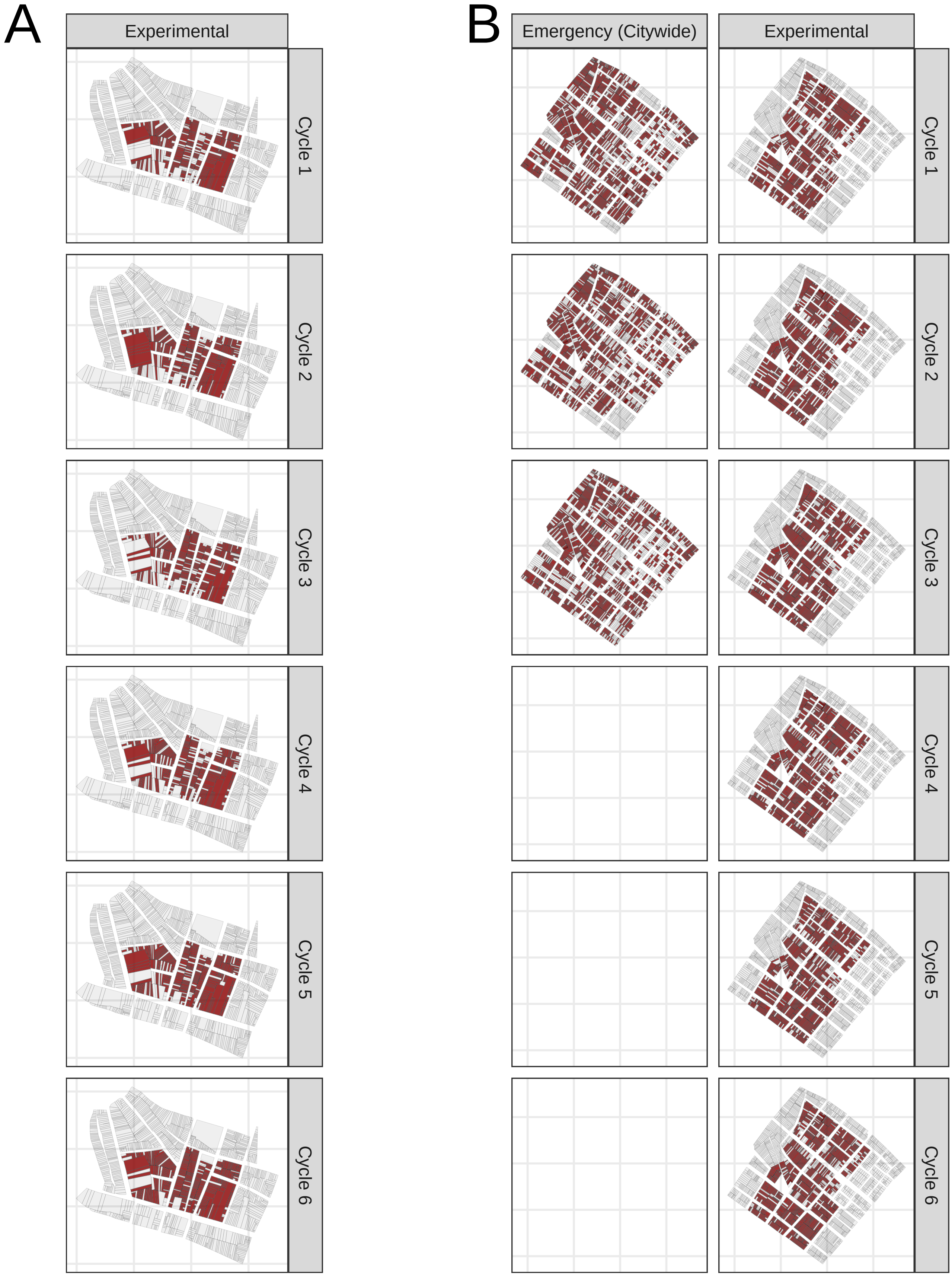


**Figure S8.** Maps of spray events (red) by spray cycle (rows). **A**: S-2013. **B**: L-2014. During L-2014, in addition to experimental spraying, 3 cycles of emergency citywide spraying were conducted. Note the map scale differs between **A** and **B**. See also Fig. 1.
