# Supplementary material for: Efficacy of Aedes aegypti control by indoor Ultra Low Volume (ULV) insecticide spraying in Iquitos, Peru
Source: PLoS Negl Trop Dis. 2018 Apr 6;12(4):e0006378. doi: 10.1371/journal.pntd.0006378 (PMC5906025; doi:10.1371/journal.pntd.0006378)
Supplement: S1 Table — Weeks: Week number from experiment start. Houses: number of unique houses surveyed. Surveys: total surveys (either adult, or combined adults and immature). Full surveys: surveys where both adult and immatures were surveyed. Buffer, Spray: surveys in buffer and spray sector, respectively. (PDF) [file pntd.0006378.s010.pdf]

Experiment	Circuit	Weeks	Treatment	Houses	Surveys	Full Surveys	Buffer	Spray
S-2013	C1	01-04		943	944	863	613	331
S-2013	C2	03-07	Exper. spray	679	983	0	603	380
S-2013	C3	09-12		935	949	885	618	331
S-2013	C4	13-16		930	967	882	614	353
L-2014	C1	01-04		1470	1473	1289	729	744
L-2014	C2	04-05		430	430	0	203	227
L-2014	C3	05-06	Citywide spray	792	848	0	411	437
L-2014	C4	07-12		1452	1500	1359	704	796
L-2014	C5	15-16		1206	1212	0	567	645
L-2014	C6	17-21	Exper. spray	1646	2502	0	1202	1300
L-2014	C7	22-27		1287	1319	1147	610	709
L-2014	C8	29-33		1461	1482	1267	720	762
L-2014	C9	41-44		1339	1358	1125	664	694

Table S1. Observation counts by Circuit. *Weeks*: Week number from experiment start. *Houses*: number of unique houses surveyed. *Surveys*: total surveys (either adult, or combined adult and immature). *Full Surveys*: surveys where both adult and immatures were surveyed. *Buffer, Spray*: surveys in buffer and spray sector, respectively.
