# Supplementary material for: Efficacy of Aedes aegypti control by indoor Ultra Low Volume (ULV) insecticide spraying in Iquitos, Peru
Source: PLoS Negl Trop Dis. 2018 Apr 6;12(4):e0006378. doi: 10.1371/journal.pntd.0006378 (PMC5906025; doi:10.1371/journal.pntd.0006378)
Supplement: S2 Table — Ratio of AA/HSE in spray sector relative to buffer sector (spray/buffer). Bold p-values: significant difference between sectors. In both years, the spray sector starts with more adults per house, and spraying reduces AA/HSE relative to buffer sectors. As in S3 Table, the effects of spraying are more pronounced in S-2013. See also Fig 4A. (PDF) [file pntd.0006378.s011.pdf]

Experiment	Circuit	Weeks	Treatment	Ratio	SE	p.value
S-2013	C1	01-04		1.52	0.31	**0.0395**
S-2013	C2	03-07	Exper. spray	0.26	0.07	**4.16e-07**
S-2013	C3	09-12		0.41	0.09	**2.19e-05**
S-2013	C4	13-16		0.84	0.16	0.357
L-2014	C1	01-04		1.24	0.16	0.09
L-2014	C2	04-05		0.90	0.21	0.659
L-2014	C3	05-06	Citywide spray	0.82	0.15	0.284
L-2014	C4	07-12		0.91	0.12	0.474
L-2014	C5	15-16		1.54	0.23	**0.0034**
L-2014	C6	17-21	Exper. spray	0.67	0.07	**0.00028**
L-2014	C7	22-27		0.71	0.10	**0.0132**
L-2014	C8	29-33		1.91	0.24	**2.72e-07**
L-2014	C9	41-44		2.53	0.34	**2.67e-12**

Table S2. Comparison between sectors (within time): Ratio of AA/HSE in spray sector relative to buffer sector (spray/buffer). **Bold p.values**: significant difference between sectors. In both years, the spray sector starts with more adults per house, and spraying reduces AA/HSE relative to buffer sectors. As in Table S3, the effects of spraying are most pronounced in 2013. See also Fig. 4A.

Experiment	Circuit	Weeks	Treatment	Ratio	SE	p.value
S-2013	C2	03-07	Exper. spray	0.17	0.05	**1.24e-09**
S-2013	C3	09-12		0.55	0.13	**0.0351**
S-2013	C4	13-16		0.89	0.20	0.944
L-2014	C2	04-05		0.85	0.16	0.979
L-2014	C3	05-06	Citywide spray	0.39	0.06	**4.87e-08**
L-2014	C4	07-12		0.69	0.09	**0.0251**
L-2014	C5	15-16		0.88	0.12	0.954
L-2014	C6	17-21	Exper. spray	0.40	0.05	**8.97e-14**
L-2014	C7	22-27		0.67	0.09	**0.0173**
L-2014	C8	29-33		1.49	0.18	**0.0103**
L-2014	C9	41-44		1.70	0.21	**0.000172**

Table S3. Comparison between times (within spray sector): Ratio of AA/HSE relative to baseline (C1, spray sector only). **Bold p.values**: significant difference from baseline circuit. In both years, spraying reduces AA/HSE relative to baseline (C1). The effects of spraying are most pronounced in S-2013, but are short-lived in both years. See also Fig. 4A.
