# Supplementary material for: Efficacy of Aedes aegypti control by indoor Ultra Low Volume (ULV) insecticide spraying in Iquitos, Peru
Source: PLoS Negl Trop Dis. 2018 Apr 6;12(4):e0006378. doi: 10.1371/journal.pntd.0006378 (PMC5906025; doi:10.1371/journal.pntd.0006378)
Supplement: S4 Table — Ratio of AA/HSE in houses that were either sprayed or not sprayed prior to surveying (no prior spray/prior spray). Bold p-values: In L-2014, houses without prior spraying yielded significantly more adults than houses with prior spraying. In S-2013, most houses were sprayed in the prior week. In L-2014, the exact date of spraying was uncertain for a small number of houses. See S5 Table for details. (PDF) [file pntd.0006378.s013.pdf]

Experiment	Contrast	Ratio	SE	p.value
S-2013	No Prior Spray / Prior Spray	1.58	1.11	0.764
L-2014	No Prior Spray / Prior Spray	2.01	0.62	**0.0472**
L-2014	Timing Unclear / Prior Spray	0.71	0.26	0.566

Table S4. Comparison between spray status (whether house was sprayed prior to survey): Ratio of AA/HSE in houses that were either sprayed or not spray prior to surveying (no prior spray / prior spray). **Bold p.values**: In L-2014, houses without prior spraying yielded significantly more adults than houses with prior spraying. In S-2013, most houses were sprayed in the prior week. In L-2014, the exact date of spraying was uncertain for a small number of houses. See Table S5 for details.

Experiment	Spray.status	nObs	Group	Est	SE	95% CI
S-2013	Prior Spray	311	b	0.06	0.02	0.03-0.14
S-2013	No Prior Spray	59	ab	0.10	0.06	0.02-0.54
L-2014	Prior Spray	889	a	0.28	0.04	0.19-0.40
L-2014	No Prior Spray	205	a	0.56	0.15	0.27-1.15
L-2014	Timing Unclear	164	ab	0.20	0.07	0.08-0.48

Table S5. Effect of prior spray on AA/HSE. A single model (NB-GLM) includes both experiment year and spray status as predictors. *Group:* significance groups (Tukey HSD) compare among all rows. Only house surveys in the spray sector during experimental spraying are included (i.e., S-2013 C2 and L-2014 C6). Not all sprayed houses were subsequently surveyed. The average interval between each house's spray application and survey was shorter in L-2014 (median 2 days) than S-2013 (median 7 days). See also Table S4.
