# Supplementary material for: Efficacy of Aedes aegypti control by indoor Ultra Low Volume (ULV) insecticide spraying in Iquitos, Peru
Source: PLoS Negl Trop Dis. 2018 Apr 6;12(4):e0006378. doi: 10.1371/journal.pntd.0006378 (PMC5906025; doi:10.1371/journal.pntd.0006378)
Supplement: S6 Table — (A) S-2013. (B) L-2014. Model estimates by circuit and treatment sector. Horizontal line separates treatment sectors; significance groups (Tukey HSD) compare among all rows. See Fig 4A for model description. (PDF) [file pntd.0006378.s015.pdf]

Circuit	Weeks	Treatment	Sector	nObs	Group	Est	SE	95% CI
C1	01-04		Buffer	613	ab	0.26	0.03	0.19-0.37
C2	03-07		Buffer	603	ab	0.26	0.03	0.18-0.37
C3	09-12		Buffer	618	c	0.53	0.06	0.39-0.72
C4	13-16		Buffer	614	a c	0.42	0.05	0.31-0.58
C1	01-04		Spray	331	abc	0.40	0.06	0.26-0.61
C2	03-07	Exper. spray	Spray	380	d	0.07	0.02	0.04-0.13
C3	09-12		Spray	331	b	0.22	0.04	0.13-0.35
C4	13-16		Spray	353	abc	0.35	0.06	0.23-0.54

Table S6A. *Ae. aegypti* adults per house (AA/HSE), 2013. Model estimates by circuit and treatment sector. Horizontal line separates treatment sectors; significance groups (Tukey HSD) compare among all rows. See Fig. 4A for model description.

Circuit	Weeks	Treatment	Sector	nObs	Group	Est	SE	95% CI
C1	01-04		Buffer	729	abcd	0.62	0.06	0.47-0.81
C2	04-05		Buffer	203	abcdef	0.72	0.12	0.43-1.21
C3	05-06	Citywide spray	Buffer	411	a gh	0.37	0.05	0.25-0.55
C4	07-12		Buffer	704	abcd	0.58	0.05	0.44-0.77
C5	15-16		Buffer	567	abc gh	0.44	0.05	0.31-0.61
C6	17-21		Buffer	1202	a c g	0.46	0.03	0.36-0.57
C7	22-27		Buffer	610	b d	0.72	0.07	0.54-0.97
C8	29-33		Buffer	720	abcd	0.60	0.06	0.45-0.79
C9	41-44		Buffer	664	abcd g	0.52	0.05	0.38-0.69
C1	01-04		Spray	744	de	0.77	0.07	0.59-1.00
C2	04-05		Spray	227	abcde	0.65	0.11	0.40-1.07
C3	05-06	Citywide spray	Spray	437	gh	0.30	0.04	0.20-0.45
C4	07-12		Spray	796	abcd g	0.53	0.05	0.40-0.69
C5	15-16		Spray	645	bcd	0.67	0.07	0.50-0.90
C6	17-21	Exper. spray	Spray	1300	h	0.31	0.02	0.24-0.39
C7	22-27		Spray	709	abcd g	0.51	0.05	0.39-0.68
C8	29-33		Spray	762	ef	1.14	0.10	0.89-1.47
C9	41-44		Spray	694	f	1.31	0.12	1.01-1.70

Table S6B. *Ae. aegypti* adults per house (AA/HSE), 2014. See Table S6A for details.
