# Supplementary material for: Efficacy of Aedes aegypti control by indoor Ultra Low Volume (ULV) insecticide spraying in Iquitos, Peru
Source: PLoS Negl Trop Dis. 2018 Apr 6;12(4):e0006378. doi: 10.1371/journal.pntd.0006378 (PMC5906025; doi:10.1371/journal.pntd.0006378)
Supplement: S7 Table — (A) S-2013. (B) L-2014. Model estimates by circuit and treatment sector. Horizontal line separates treatment sectors, significance groups (Tukey HSD) compare among all rows. See Fig 4B for model description. (PDF) [file pntd.0006378.s016.pdf]

Circuit	Weeks	Treatment	Sector	nObs	Group	Est	SE	95% CI
C1	01-04		Buffer	613	ab	0.15	0.01	0.11-0.19
C2	03-07		Buffer	603	ab	0.15	0.01	0.11-0.19
C3	09-12		Buffer	618	a c	0.21	0.02	0.17-0.26
C4	13-16		Buffer	614	c	0.23	0.02	0.19-0.28
C1	01-04		Spray	331	abc	0.16	0.02	0.11-0.22
C2	03-07	Exper. spray	Spray	380	d	0.06	0.01	0.03-0.10
C3	09-12		Spray	331	b	0.13	0.02	0.08-0.19
C4	13-16		Spray	353	abc	0.17	0.02	0.12-0.23

Table S7A. Proportion *Ae. aegypti* adult-infested houses (PrIH), 2013. Model estimates by circuit and treatment sector. Horizontal line separates treatment sectors; significance groups (Tukey HSD) compare among all rows. See Fig. 4B for model description.

Circuit	Weeks	Treatment	Sector	nObs	Group	Est	SE	95% CI
C1	01-04		Buffer	729	ab	0.31	0.02	0.26-0.36
C2	04-05		Buffer	203	abcd	0.33	0.03	0.24-0.43
C3	05-06	Citywide spray	Buffer	411	ef	0.16	0.02	0.11-0.22
C4	07-12		Buffer	704	abc g	0.26	0.02	0.22-0.32
C5	15-16		Buffer	567	c e g	0.22	0.02	0.17-0.28
C6	17-21		Buffer	1202	c e g	0.21	0.01	0.18-0.25
C7	22-27		Buffer	610	abc g	0.27	0.02	0.22-0.33
C8	29-33		Buffer	720	c e g	0.22	0.02	0.18-0.27
C9	41-44		Buffer	664	abc g	0.26	0.02	0.21-0.32
C1	01-04		Spray	744	a d	0.34	0.02	0.29-0.39
C2	04-05		Spray	227	abcd g	0.29	0.03	0.21-0.39
C3	05-06	Citywide spray	Spray	437	e g	0.18	0.02	0.13-0.24
C4	07-12		Spray	796	bc e g	0.23	0.01	0.19-0.28
C5	15-16		Spray	645	abc	0.28	0.02	0.23-0.33
C6	17-21	Exper. spray	Spray	1300	f	0.11	0.01	0.09-0.14
C7	22-27		Spray	709	c e g	0.22	0.02	0.18-0.27
C8	29-33		Spray	762	a d	0.34	0.02	0.29-0.40
C9	41-44		Spray	694	d	0.41	0.02	0.36-0.47

Table S7B. Proportion *Ae. aegypti* adult-infested houses (PrIH), 2014. See Table S7A for details.
