# Supplementary material for: Efficacy of Aedes aegypti control by indoor Ultra Low Volume (ULV) insecticide spraying in Iquitos, Peru
Source: PLoS Negl Trop Dis. 2018 Apr 6;12(4):e0006378. doi: 10.1371/journal.pntd.0006378 (PMC5906025; doi:10.1371/journal.pntd.0006378)
Supplement: S8 Table — (A) S-2013. (B) L-2014. Model estimates by circuit and treatment sector. Horizontal line separates treatment sectors, significance groups (Tukey HSD) compare among all rows. See S5 Fig. (PDF) [file pntd.0006378.s017.pdf]

Circuit	Weeks	Treatment	Sector	nObs	Group	Est	SE	95% CI
C1	01-04		Buffer	58	a	0.11	0.04	0.05-0.25
C2	03-07		Buffer	52	a	0.10	0.03	0.04-0.22
C3	09-12		Buffer	73	a	0.11	0.03	0.05-0.21
C4	13-16		Buffer	92	a	0.16	0.03	0.09-0.28
C1	01-04		Spray	32	a	0.23	0.06	0.10-0.43
C2	03-07	Exper. spray	Spray	9	a	0.09	0.09	0.01-0.64
C3	09-12		Spray	23	a	0.04	0.04	0.00-0.37
C4	13-16		Spray	34	a	0.18	0.05	0.08-0.37

Table S8A. Proportion nulliparouous *Ae. aegypti* females (PrNF), 2013. Model estimates by circuit and treatment sector. Horizontal line separates treatment sectors; significance groups (Tukey HSD) compare among all rows. See also Fig. S5.

Circuit	Weeks	Treatment	Sector	nObs	Group	Est	SE	95% CI
C1	01-04		Buffer	144	a	0.03	0.01	0.01-0.09
C2	04-05		Buffer	3	ab	0.33	0.27	0.01-0.95
C3	05-06	Citywide spray	Buffer	39	ab	0.10	0.04	0.03-0.29
C4	07-12		Buffer	120	a	0.07	0.02	0.03-0.14
C5	15-16		Buffer	76	ab	0.06	0.02	0.02-0.17
C6	17-21		Buffer	155	a	0.06	0.01	0.03-0.12
C7	22-27		Buffer	93	ab	0.08	0.02	0.04-0.16
C8	29-33		Buffer	95	a	0.05	0.01	0.02-0.11
C9	41-44		Buffer	93	a	0.03	0.01	0.01-0.12
C1	01-04		Spray	155	a	0.07	0.02	0.03-0.13
C2	04-05		Spray	39	ab	0.03	0.02	0.00-0.21
C3	05-06	Citywide spray	Spray	47	ab	0.11	0.04	0.04-0.29
C4	07-12		Spray	106	ab	0.12	0.02	0.06-0.21
C5	15-16		Spray	101	ab	0.09	0.02	0.04-0.17
C6	17-21	Exper. spray	Spray	95	b	0.20	0.03	0.13-0.31
C7	22-27		Spray	96	ab	0.10	0.02	0.05-0.19
C8	29-33		Spray	161	a	0.05	0.01	0.02-0.09
C9	41-44		Spray	197	ab	0.11	0.01	0.07-0.16

Table S8B. Proportion nulliparouous *Ae. aegypti* females (PrNF), 2014. See Table S8A for details.
