# Supplementary material for: Efficacy of Aedes aegypti control by indoor Ultra Low Volume (ULV) insecticide spraying in Iquitos, Peru
Source: PLoS Negl Trop Dis. 2018 Apr 6;12(4):e0006378. doi: 10.1371/journal.pntd.0006378 (PMC5906025; doi:10.1371/journal.pntd.0006378)
Supplement: S9 Table — (A) S-2013. (B) L-2014. Note that Breteau Index (BI) = 100*PC/HSE. Model estimates by circuit and treatment sector. Horizontal line separates treatment sectors, significance groups (Tukey HSD) compare among all rows. See S5 Fig. (PDF) [file pntd.0006378.s018.pdf]

Circuit	Weeks	Sector	nObs	Group	Est	SE	95% CI
C1	01-04	Buffer	565	a	0.09	0.02	0.060-0.147
C3	09-12	Buffer	590	ab	0.16	0.02	0.111-0.234
C4	13-16	Buffer	583	ab	0.15	0.02	0.103-0.221
C1	01-04	Spray	297	ab	0.09	0.02	0.051-0.175
C3	09-12	Spray	282	a	0.07	0.02	0.038-0.148
C4	13-16	Spray	268	b	0.22	0.04	0.134-0.373

Table S9A. *Ae. aegypti* Positive Containers per House (PC/HSE), 2013. Breteau index (BI) = 100× PC/HSE. Model estimates by circuit and treatment sector. Horizontal line separates treatment sectors; significance groups (Tukey HSD) compare among all rows. No container surveys were conducted during spraying. See also Fig. S5.

Circuit	Weeks	Sector	nObs	Group	Est	SE	95% CI
C1	01-04	Buffer	638	abc	0.10	0.02	0.063-0.159
C4	07-12	Buffer	606	abc	0.08	0.01	0.048-0.131
C7	22-27	Buffer	514	a	0.15	0.02	0.095-0.237
C8	29-33	Buffer	629	bc	0.06	0.01	0.034-0.102
C9	41-44	Buffer	564	b	0.04	0.01	0.023-0.084
C1	01-04	Spray	649	abc	0.10	0.02	0.064-0.158
C4	07-12	Spray	710	abc	0.08	0.01	0.052-0.132
C7	22-27	Spray	613	a c	0.12	0.02	0.076-0.186
C8	29-33	Spray	621	bc	0.06	0.01	0.037-0.108
C9	41-44	Spray	551	abc	0.08	0.01	0.045-0.130

Table S9B. *Ae. aegypti* Positive Containers per House (PC/HSE), 2014. Breteau index (BI) = 100× PC/HSE. See Table S9A for details.
