# Supplementary material for: Efficacy of Aedes aegypti control by indoor Ultra Low Volume (ULV) insecticide spraying in Iquitos, Peru
Source: PLoS Negl Trop Dis. 2018 Apr 6;12(4):e0006378. doi: 10.1371/journal.pntd.0006378 (PMC5906025; doi:10.1371/journal.pntd.0006378)
Supplement: S10 Table — (A) S-2013. (B) L-2014. Model estimates by circuit and treatment sector. Horizontal line separates treatment sectors, significance groups (Tukey HSD) compare among all rows. No container surveys were conducted during spraying See also S5 Fig. (PDF) [file pntd.0006378.s019.pdf]

Circuit	Weeks	Sector	nObs	Group	Est	SE	95% CI
C1	01-04	Buffer	565	a	0.04	0.00	0.026-0.053
C3	09-12	Buffer	590	b	0.06	0.01	0.047-0.079
C4	13-16	Buffer	583	ab	0.06	0.01	0.045-0.077
C1	01-04	Spray	297	ab	0.04	0.01	0.023-0.062
C3	09-12	Spray	282	ab	0.04	0.01	0.024-0.073
C4	13-16	Spray	268	c	0.11	0.01	0.076-0.145

Table S10A. Proportion *Ae. aegypti* Positive Containers (PrPC), 2013. Model estimates by circuit and treatment sector. Horizontal line separates treatment sectors; significance groups (Tukey HSD) compare among all rows. No container surveys were conducted during spraying. See also Fig. S5.

Circuit	Weeks	Sector	nObs	Group	Est	SE	95% CI
C1	01-04	Buffer	638	abc	0.05	0.01	0.033-0.066
C4	07-12	Buffer	606	ab	0.04	0.01	0.026-0.057
C7	22-27	Buffer	514	c	0.07	0.01	0.053-0.098
C8	29-33	Buffer	629	a	0.03	0.00	0.019-0.048
C9	41-44	Buffer	564	a	0.02	0.00	0.013-0.040
C1	01-04	Spray	649	abc	0.04	0.01	0.031-0.061
C4	07-12	Spray	710	ab	0.04	0.00	0.026-0.053
C7	22-27	Spray	613	bc	0.06	0.01	0.044-0.082
C8	29-33	Spray	621	ab	0.03	0.01	0.022-0.053
C9	41-44	Spray	551	abc	0.04	0.01	0.027-0.063

Table S10B. Proportion *Ae. aegypti* Positive Containers (PrPC), 2014. See Table S10A for details.
