# Supplementary material for: Efficacy of Aedes aegypti control by indoor Ultra Low Volume (ULV) insecticide spraying in Iquitos, Peru
Source: PLoS Negl Trop Dis. 2018 Apr 6;12(4):e0006378. doi: 10.1371/journal.pntd.0006378 (PMC5906025; doi:10.1371/journal.pntd.0006378)
Supplement: S1 Text — (PDF) [file pntd.0006378.s020.pdf]

Text S1: Spraying Details.

Spraying was done in the evening between 17:00-20:00 to avoid high temperatures and varying winds. Spray operators began the application at the back of the house (backyard), moving through the home until they reached the front door. The operator would apply insecticide for a maximum of 15 seconds in each room. All windows and doors were closed as the team finished.

Two spray teams were typically assigned to each block of houses designated for spraying. The two spray teams would start at neighboring houses and proceed in opposite directions until they met on the opposite side of the block, thus visiting all accessible houses on that block. Each spray team would circle its assigned block at least twice to maximize the chance of finding residents at home. The monitor recorded which houses were and were not sprayed, and the reasons why houses were not sprayed (i.e., closed, vacant lot, unoccupied, refused). If a house's occupants refused to participate, the reason was recorded (including no time, sick individual present, did not believe that spraying works, etc.). On subsequent days, spray teams were provided a list of houses that had not been previously sprayed so they could revisit these houses, except in cases of complete refusal. These previously unsprayed houses were revisited daily when logistically feasible (from 3-10 visits, until Friday, if needed) in an attempt to conduct spraying.

The MOH typically uses backpack sprayers because of their ease of use and durability. During S-2013, however, we collaborated with MOH teams to test the Colt sprayers during cycles 4, 5 and 6, because the Colt sprayers are generally thought to provide more consistent

droplet size. Because only 2 Colt sprayers were available, only 1/3rd of the houses could be treated with the Colt sprayers on Mondays. There was a total of 6 spray teams. During the S-2013 and L-2014 experiments, only Colt sprayers were used to spray revisited houses that were missed on the first spray day.

During the L-2014 emergency spraying, the MOH provided us with anticipated spray schedules and our monitors accompanied MOH spray teams to record which houses were sprayed. On a few blocks our monitors arrived after the MOH spray teams, but verified spraying with residents.
