# Supplementary material for: Efficacy of Aedes aegypti control by indoor Ultra Low Volume (ULV) insecticide spraying in Iquitos, Peru
Source: PLoS Negl Trop Dis. 2018 Apr 6;12(4):e0006378. doi: 10.1371/journal.pntd.0006378 (PMC5906025; doi:10.1371/journal.pntd.0006378)
Supplement: S2 Text — (PDF) [file pntd.0006378.s021.pdf]

**Text S2: Sampling Details**

In some weeks during L-2014, the exact timing of each house spray was not available. Therefore, the interval between spray and subsequent survey is uncertain for a minority of surveys (Tables S4 and S5). For the majority of L-2014 house surveys, however, sprays occurred within 2 days of spraying, and all house surveys within the spray period occurred within 4 days of spraying. Thus, the average interval between house spray and survey was shorter in L-2014 compared with S-2013 (median 2 days and 7 days, respectively). In L-2014, significantly more adults were found in Spray Sector houses that had not been sprayed prior to surveying, compared to houses that had been previously sprayed (Tables S4 and S5).

The original S-2013 design called for surveys in both the spray and buffer sectors to take place concurrently. Due to logistical constraints, this design was not always followed. In the beginning of S-2013, timing of the baseline circuit of the buffer sector (C1) overlapped with the subsequent spraying circuit of the spray sector (C2, Fig 3A).

Due to logistic constraints in both S-2013 and L-2014, several circuits were not sampled in a spatially systematic manner. Instead, surveys in these subcircuits were conducted in spatially coherent set of blocks. These circuits included the pre-intervention Circuit 1 of both experiments, the emergency pre-MOH Circuit 2 of L-2014, and the post-intervention Circuit 7 of L-2014 (Fig S7).
